# The Tree of Life and a New Classification of Bony Fishes

**DOI:** 10.1371/currents.tol.53ba26640df0ccaee75bb165c8c26288

**Published:** 2013-04-18

**Authors:** Ricardo Betancur-R., Richard E. Broughton, Edward O. Wiley, Kent Carpenter, J. Andrés López, Chenhong Li, Nancy I. Holcroft, Dahiana Arcila, Millicent Sanciangco, James C Cureton II, Feifei Zhang, Thaddaeus Buser, Matthew A. Campbell, Jesus A Ballesteros, Adela Roa-Varon, Stuart Willis, W. Calvin Borden, Thaine Rowley, Paulette C. Reneau, Daniel J. Hough, Guoqing Lu, Terry Grande, Gloria Arratia, Guillermo Ortí

**Affiliations:** The George Washington University; University of Oklahoma; Biologist at The University of KansasUniversity of Kansas; International Union for Conservation of NatureOld Dominion University; University of Alaska MuseumUniversity of Alaska Fairbanks; Shanghai Ocean University; Johnson County Community College; The George Washington University; Old Dominion University; University of Oklahoma; Ph.D. studentUniversity of Oklahoma; University of Alaska Fairbanks; The George Washington University; Research AssistantGeorge Washington University; University of Nebraska-Lincoln; Loyola University Chicago; University of Nebraska-Omaha; Florida A&M University; University of Oklahoma; University of Nebraska at Omaha; Loyola University Chicago; Courtesy Research Professor and Associated ResearcherUniversity of Kansas; The George Washington University

## Abstract

The tree of life of fishes is in a state of flux because we still lack a comprehensive phylogeny that includes all major groups. The situation is most critical for a large clade of spiny-finned fishes, traditionally referred to as percomorphs, whose uncertain relationships have plagued ichthyologists for over a century. Most of what we know about the higher-level relationships among fish lineages has been based on morphology, but rapid influx of molecular studies is changing many established systematic concepts. We report a comprehensive molecular phylogeny for bony fishes that includes representatives of all major lineages. DNA sequence data for 21 molecular markers (one mitochondrial and 20 nuclear genes) were collected for 1410 bony fish taxa, plus four tetrapod species and two chondrichthyan outgroups (total 1416 terminals). Bony fish diversity is represented by 1093 genera, 369 families, and all traditionally recognized orders. The maximum likelihood tree provides unprecedented resolution and high bootstrap support for most backbone nodes, defining for the first time a global phylogeny of fishes. The general structure of the tree is in agreement with expectations from previous morphological and molecular studies, but significant new clades arise. Most interestingly, the high degree of uncertainty among percomorphs is now resolved into nine well-supported supraordinal groups. The order Perciformes, considered by many a polyphyletic taxonomic waste basket, is defined for the first time as a monophyletic group in the global phylogeny. A new classification that reflects our phylogenetic hypothesis is proposed to facilitate communication about the newly found structure of the tree of life of fishes. Finally, the molecular phylogeny is calibrated using 60 fossil constraints to produce a comprehensive time tree. The new time-calibrated phylogeny will provide the basis for and stimulate new comparative studies to better understand the evolution of the amazing diversity of fishes.

## Introduction

“…With the variety of both primitive and advanced teleosts living today, we are most emphatically of the opinion that approaches other than morphological ones would be exceedingly fruitful in the investigation of teleostean interrelationships..."

Greenwood et al. (1966)^1^

Our view of the phylogeny and classification of bony fishes is rapidly changing under the influence of molecular phylogenetic studies based on larger and more taxonomically comprehensive datasets. Classification schemes displayed in widely used text books on fish biodiversity (e.g.,^2^^,^ 3) have been based on loosely formulated syntheses (supertrees) and community consensus views of largely disconnected studies. The phylogenetic structure underpinning such classifications has many areas that are notably unresolved and poorly known, providing weak or no justification for many groups that, although formally recognized, are implicitly known to be polyphyletic (e.g. percoids, perciforms, scorpaeniforms). A comprehensive phylogenetic tree for all major groups of fishes has been elusive because explicit analyses including representatives across their diversity have never been accomplished. Detailed morphological cladistic investigations of fish relationships have typically focused on lower taxonomic scales and few attempts to synthesize morphology at higher taxonomic levels proved to be challenging and met limited success (e.g.,^4^). A recent effort to systematically collect morphological synapomorphies from published records for all currently recognized groups resulted in the first teleost classification based on monophyletic groups^5^. This effort, however, did not produce a global phylogenetic hypothesis. Similarly, molecular analyses have been limited and many times conflicting in terms of genetic coverage and taxonomic sampling.

As predicted by Greenwood et al.^1^, development of molecular markers, especially sequences of mitochondrial DNA (mtDNA) genes or complete mitochondrial genomes, catalyzed new views of bony fish relationships by providing a common yardstick of phylogenetic information across vast taxonomic scales^6^^,^ 7 ^,^ 8. Studies based on mitogenomic data proliferated to methodically probe conflicting hypotheses of relationship for several groups at diverse taxonomic levels, many times proposing alternative arrangements supporting new clades unsuspected by previous classifications^10^^,^ 11 ^,^ 12 ^,^ 13. In spite of their new powerful insights, mitogenomic hypotheses were not universally embraced because they represent information from a single locus, prompting corroboration from additional genomic regions. Several nuclear DNA markers were subsequently developed and applied to infer bony fish relationships. The most popular ones include 28S ribosomal subunit^14^^,^ 15 ^,^ 16, *tmo4c4* 17 ^,^ 18, rhodopsin^19^^,^ 20, *rag1* and *rag2* 21 ^,^ 22 *, mll* 20, *irbp* 23, and *rnf213* 24. Using a systematic approach to scan genomic databases, a larger set of nuclear markers became available in 2007 25, opening a new window to obtaining large multilocus datasets^25^^,^ 26 ^,^ 27 ^,^ 28 ^,^ 66. Recent studies using between 10 and 20 of these nuclear markers for a few hundred taxa^27^^,^ 28 ^,^ 29 ^,^ 30 ^,^ 31 ^,^ 66, have shown improved resolution of phylogenetic relationship at higher and lower taxonomic levels. Many but not all of the mitogenomic hypotheses received support from nuclear gene data, but the discovery of new clades continued with increasing taxonomic sampling. Initially identified by letters (e.g., clades A, B, C, etc.^19^^,^ 23 ^,^ 32), new names were recently proposed for many groupings supported by molecular evidence, such as Stiassnyiformes, Zeiogadiformes, Carangimorpha, Cottimorpha, Ovalentaria, Gobiiformes etc.^24^^,^ 31 ^,^ 33. Validation of these groups (and their proposed names) is pending until a comprehensive study including all taxa is produced. Proliferation of new names is useful for identification of the newly discovered groups, but may create confusion if not systematically organized into a global classification.

Molecular phylogenetic methods (e.g., BEAST^34^) in combination with fossil evidence also opened a new temporal window to understand bony fish diversification. Attempts to estimate divergence dates among crow-group lineages using this approach (e.g., 35 ^,^ 36 ^,^ 37) frequently produced conflicting views with the paleontological literature^38^^,^ 39 ^,^ 40, sometimes implying large gaps in the fossil record. The discrepancy is larger when divergence estimates for crown teleost lineages have been based on mitogenomic data (e.g.,^37^^,^ 41 ^,^ 115). Nucleotide saturation, compressing basal branch lengths for mtDNA, and the specific approaches used to apply fossils constraints to calibrate the molecular phylogeny may explain this discordance^43^. Other studies based on several nuclear genes and larger sets of fossil calibration points produced divergence dates more consistent with the fossil record^29^^,^ 66, but a comprehensive time-tree for osteichthyan diversification is not yet available.

The shape of the bony fish tree of life is currently better resolved for the early-branching lineages than for the more apical acanthomorph groups, in particular the percomorphs, a large and diverse group of spiny-finned fishes with uncertain affinities that came to be known as **“**bush at the top**”** 44. Few basal branching events among osteichthyans remain problematic, for example, the relationships among lungfishes, coelacanths, and tetrapods^45^^,^ 46 ^,^ 47 ^,^ 66. In contrast, the basal branching pattern for early extant actinopterygians (involving polypteriforms, chondrosteans, lepisosteids, *Amia* and teleosts) have been resolved with confidence based on morphological and DNA sequence evidence^66^. Similarly, recent molecular studies based on several nuclear genes^25^ consistently support relationships among major teleost groups: Elopomorpha, Osteoglossomorpha and Euteleostei^29^^,^ 66. The deeper nodes among euteleosts and percomorphs also could be resolved with confidence with this new set of nuclear markers, but a comprehensive phylogeny including all groups is lacking. In this study we report phylogenetic results based on a taxonomically comprehensive dataset with DNA sequences for 21 nuclear genes. A dataset with 1416 taxa was assembled, including four tetrapod and two chondrichthyan outgroups. Bony fish diversity is represented by 1093 genera (of ca. 4300), 369 families (of 502), and all traditionally recognized orders^5^, making this the most comprehensive dataset ever compiled in systematic ichthyology. Phylogenetic results corroborate many previously established hypotheses, but also provide unprecedented resolution among percomorphs. The uncertain relationships involving most of the extant diversity of percomorphs is resolved into several well-supported groups and, for the first time, we offer a monophyletic definition for Perciformes. Using a set of 60 calibrations, we also provide the most comprehensive hypothesis to date about the tempo of osteichthyan diversification. Considering the new clades obtained in this study and previously published well-supported clades, we propose a new classification for bony fishes based on the nomenclatural scheme recently proposed by Wiley and Johnson^5^. Our hope is that this explicit proposal will facilitate communication among ichthyologists attempting to chart the rapidly changing landscape of phylogeny and classification of fishes.

## Materials and Methods

**Molecular data and taxonomic sampling**

This study is the main product of the Euteleost Tree of Life Project (EToL). A total of 21 molecular markers with a genome-wide distribution were examined, the majority of which were developed by EToL using a genomic screen pipeline^25^. This pipeline compared the *Danio rerio* and *Takifugu rubripes* genomes to identify single-copy genes with long exons (>800 bp) and divergence levels suggesting they evolve at rates appropriate for phylogenetic resolution among distantly related taxa. Exons markers were sequenced from 11 nuclear genes previously published by our group (*kiaa1239*, *ficd*, *myh6*, *panx2*, *plagl2*, *ptchd4* (=*ptr*), *ripk4*, *sidkey*, *snx33* (=*sh3px3*), *tbr1b* (=*tbr1*), and *zic1*) and three additional markers, including one intron (*hoxc6a*) and two exons (*svep1*, and *vcpip*), were newly developed for this study using the same approach. Sequence data from seven additional markers, including EToL markers (*enc1*, *gtdc2* (=*glyt*), and *gpr85* (=*sreb2*)) or markers developed by others (16S mtDNA, *rag1*, *rag2*, and *rh*), were generated for our previous studies (e.g., 25 ^,^ 26 ^,^ 27 ^,^ 28 ^,^ 66 ^,^ 96) or obtained from NCBI, Ensembl, or other genomic databases.

A total of 1184 bony fish taxa were initially targeted for this study and samples were primarily obtained from the tissue repository of the Ichthyology Collection at University of Kansas (1129 samples) or other collections. Of the initial list, samples for 18 taxa either failed to amplify or belonged to duplicate species that were ultimately combined or discarded. Sixty taxa that produced sequence data for one or two genes only were also discarded. Twenty-five additional taxa were excluded from the final matrix because they had low genetic coverage and highly variable phylogenetic placement in preliminary analyses, as identified using bootstrap trees obtained with RAxML v7.3^49^ and the RogueNaRok server 50. Our final sampling thus included 1081 taxa and sequence data from 335 additional taxa were obtained from previous EToL studies (e.g., 25 ^,^ 26 ^,^ 27 ^,^ 28 ^,^ 66 ^,^ 96) or public databases (Table S1). In order to minimize missing data, some sequences retrieved from public databases were combined as genus-level composite taxa (52 taxa). DNA extraction, amplification protocols via nested PCR, and primers followed previous studies (e.g., 25 ^,^ 26 ^,^ 27 ^,^ 28 ^,^ 66 ^,^ 96). Primer sequences and optimized PCR conditions used for the three new markers is presented in Table 1. The PCR amplicons obtained were submitted for purification and sequencing in both directions to High Throughput Sequencing Solutions (HTSeq.org) or other core facilities.

Fish diversity is represented in the phylogenetic data matrix by a sample of 1410 bony fish species (of ca. 31000^51^) plus four tetrapod species and two chondrichthyan outgroups (total 1416 terminals). The taxonomic sampling of bony fishes consists of 1093 genera (of ca. 4300), 369 families (of 502; see below), and all traditionally recognized orders (e.g.,^5^). Our taxonomic sampling emphasizes representation of percomorph groups, with 1037 (of >15000) species in 201 families. All scientific names were checked against the Catalog of Fishes 51. A complete list of material examined is given in Table S1.

**Table 1. Primers used for new markers developed and optimized PCR conditions.** *1st and 2nd are primers for the first and nested/seminested (optional) rounds of PCR, respectively.

**Marker_primer name**	**Primer sequence**	**Optimized temp.**	PCR*
hoxc6a_F215	5'-ATGGATCAAACGTGTTTCTTCA-3'	60-56	1st
hoxc6a_R1129	5'-GCGATYTCGATGCGTCTGCG-3'	60-56/62-58	1st/2nd
hoxc6a_F386	5'-GATCTACCCGTGGATGCAGCG-3'	62-58	2nd
svep1_F7960	5'-CCTCCNCAYATYGAYTTTGGDGAMTA-3'	50	1st
svep1_R8889	5'-TTCAGGWARCCRTGRCTRATRTCCTC-3'	50	1st
vcpip_F84	5'-CCGGACCCGMARTGYCAGGC-3'	52	1st
vcpip_R946	5'-GTGRTTBCKGCYVGAGCTGCTCCABGC-3'	52	1st
vcpip_F134	5'-AGCATYGAGTGCACSGASTGCGGMCA-3'	52	2nd
vcpip_R930	5'-CTGCTCCASGCRATGCAKATGGGYTTG-3'	52	2nd

**Sequence alignment and phylogenetic analyses**

Contigs were assembled from forward and reverse sequences using CodonCode Aligner v3.5.4 (CodonCode Corporation), Sequencher v4 (Gene Codes Corporation), or Geneious Pro v4.5 (Biomatters Ltd.). Exon markers were aligned individually based on their underlying reading frame in TranslatorX^52^ using the MAFFT aligner^53^. The *hoxc6a* and 16S sequences were aligned with MAFFT v6.9^53^ using 1000 iterations and the genafpair algorithm. Because nested PCR is highly prone to cross-contamination, we vetted the data by visually inspecting individual gene trees estimated with the Geneious Tree Builder algorithm in Geneious. To qualitatively assess gene-tree congruence, the final gene alignments were analyzed under maximum likelihood (ML) in RAxML using ten independent runs for each; exon alignments were partitioned by codon position. Alternative approaches to analyze combined data based on species-tree methods that account for gene-tree heterogeneity due to lineage sorting (e.g.,^54^^,^ 55 ^,^ 56 ^,^ 57) could not be applied to this dataset due to high proportion of missing data (see Results).**

Individual genes were concatenated using SequenceMatrix v1.7.8^58^ or Geneious. Two datasets were assembled and analyzed separately, one including all 1416 taxa with sequence data from three genes or more (3+ dataset) and a subset including 1020 taxa with sequence data from seven genes or more (7+ dataset). Analyses of the 3+ dataset were performed under maximum likelihood (ML) using two partitioning schemes, a simple one determined arbitrarily with 5 data partitions (3 codon positions across all exons plus 16S and *hoxc6a*), and a more complex scheme with 24 partitions (a combination of codon positions and individual genes plus 16S and *hoxc6a*) indicated by PartitionFinder^59^. To make the PartitionFinder analysis scalable, a representative subset of 201 taxa was run under the Bayesian Information Criterion^59^. The 7+ dataset was analyzed with the 24-partition scheme only. Analyses for both datasets and partition schemes were conducted in RAxML using 30 independent replicates under the GTRGAMMA model. Nodal support was assessed using the rapid bootstrapping algorithm of RAxML with 1000 replicates estimated under the GTRCAT model^60^, and the collection of sample trees was used to draw the bibartition frequencies on the optimal tree. All RAxML analyses were conducted in the CIPRES portal v3.1.

For comparison purposes, the 3+ dataset was also analyzed under implied-weighted parsimony^61^. The optimal tree search and bootstrap trees were set to run independently. Gaps were treated as missing characters and all parsimony uninformative characters were ignored. A relatively mild value of *k* (20) was chosen arbitrarily due to computational limitations to explore sensitivity of the nodes to other weighting functions. Tree searches were performed in TNT 1.1^63^ using a driven-search strategy combining the following tree-search algorithms: ratchet, drift, sectorial searches and tree fusion. The exhaustiveness of the search parameters was self-adjusted every 2 hits of the current best score. To maximize tree-space exploration, the final searches implemented tree-bisection-reconnection (TBR). A strict consensus of nine equally optimal trees (length 407187 steps; fit 7309.19) was computed. Bootstrap search strategies were relaxed to ten random addition sequences and TBR, saving only one tree per replicate (1000 replicates); bootstrap bipartition frequencies were drawn on the consensus tree.

**Divergence time estimates**

Time-tree estimation in a Bayesian framework using the complete dataset was computationally infeasible. Thus, we selected a subset of 202 taxa for 18 genes that had representation of: (i) all major bony fish lineages, (ii) lineages encompassing the nodes in which the assignment of fossil calibrations is most informative, (iii) taxa with the highest genetic coverage to minimize missing data in the data matrix (the markers *vcpip*, *svep1*, *hoxc6a*, including a high proportion of missing data, were also excluded). Divergence times were estimated in BEAST v1.7 using the uncorrelated log-normal (UCLN) clock-model^34^. Sixty calibration points were selected as priors for divergence time estimates, of which 58 are based on previous studies^29^^,^ 64 ^,^ 65 ^,^ 66 and two (calibrations 45 and 60) are proposed here (Appendix 1). However, the actual BEAST analysis conducted for this study included 59 calibrations only (see details under calibration 60, Appendix 1). A starting chronogram that satisfied all priors (e.g., monophyly and initial divergence times) was generated under penalized likelihood in r8s v1.71^67^ using the RAxML tree. To model branching rates on the tree, a birth-death process was used for the tree prior with initial birth rate = 1.0 and death rate = 0.5. The substitution model was GTR+G with 4 rate classes and the data were partitioned into 4 categories with independent parameter estimation: three codon positions across exons of protein-coding genes plus 16S. Clock and tree priors were linked across partitions. Five replicates of the Markov chain Monte Carlo (MCMC) analyses were each run for 200 million generations, with the topology constrained to that recovered in the phylogenetic analyses of the 3+ dataset (pruned for taxa not included in the subset). Post-run analysis of MCMC log files was assessed using Tracer v. 1.5^68^ and mixing was considered complete if the effective sample size of each parameter was >200^34^^,^ 68. Tree files from the five runs were combined in LogCombiner v1.7.4^68^ with the first 10% of trees from each run discarded as burn-in. The maximum clade credibility tree, with means and 95% highest posterior density of divergence times, was estimated with TreeAnnotator v1.6.1^68^. **

The complete tree with 1416 taxa was time-calibrated under penalized likelihood (PL^67^) with treePL^69^. The PL model, which assumes rate autocorrelation, has been shown to perform poorly in simulation studies resulting in high stochastic error of divergence time estimates^70^. To ameliorate this problem, mean highest posterior density estimates of clade ages obtained with the subset in BEAST were imposed as fixed secondary calibrations for the PL analysis, rather than using primary calibrations with minimum and maximum age constrains. A total of 126 secondary calibrations were used for this analysis, including the ages obtained for all major groups in the tree as well as the nodes near which primary calibrations were defined. The rate smoothing parameter was set to 10 based on the cross-validation procedure and the **χ^2^** test in treePL (four smoothing values between 1 and 1000 were compared).****

## Results and Discussion

The final concatenated alignments included 21 markers with 20853 sites for 1416 taxa in the 3+ dataset and 1020 taxa in the 7+ dataset. The average presence of data (number of sequences per taxon) across the alignments was 41.0% for the 3+ dataset and 48.2% for the 7+ dataset. A summary of dataset features, including data presence, alignment length, and sequence variation for each marker is given in Table 2 (see also Table S1). The new sequences have been deposited in GenBank under accession numbers KC825360-KC831391. The sequence alignment (nexus format), ML tree (newick format), and Table S1 are available from the Dryad repository (DOI:10.5061/dryad.c4d3j). The main phylogenetic hypothesis is summarized in Fig. 1 (24-partition RAxML tree, 3+ dataset, time-calibrated under PL). Fig. 2 provides measures of congruence among alternative analyses (concatenation and gene trees) for all major clades and provides discrete tests for traditional hypotheses in ichthyology. Figs. 3–10 provide more detail on the relationships within selected percomorph clades based on the tree in Fig. 1. The time-calibrated (BEAST) tree for the subset (202 taxa and 18 genes) and 59 calibration points are shown in Fig. 11 (see also Appendix 1); Fig. 12 compares the results of divergence times estimated for major groups with those obtained by other recent multi-locus studies. The complete phylogeny with bootstrap values and taxonomic annotations is depicted in Fig. S1 as a cladogram and can also be visualized online as a time-tree using a fractal explorer and zooming interface at OneZoom 73 (also posted at DeepFin).

The basal nodes of the tree and relationships among early branching groups of bony fishes have been well established and thoroughly discussed by recent molecular systematic studies based on similar sets of genes^29^^,^ 66, albeit with reduced taxonomic sampling. Because our results corroborate these hypotheses (e.g. monophyly of Actinopterygii and Holostei, branching order of elopomorphs and osteoglossomorphs; Fig. 1), we refer the reader to those papers for discussion on relationships among lineages from the root of the tree up to the Euteleosteomorpha. The most significant new results involve crown acanthomorph lineages, in particular the unprecedented resolution among percomorphs, represented in this study by 1037 species in 201 families. The proverbial “bush at the top” is now disambiguated into several well-supported clades at the ordinal or supraordinal level, with well-resolved relationships amongst them (Fig. 1). We also provide for the first time a monophyletic definition of Perciformes, sinking into this clade components of Scorpaeniformes, Gasterosteiformes, and Cottiformes (Fig. 10; see also^16^^,^ 74). Among the euacanthomorphs, we find the non-monophyly of Beryciformes (including Stephanoberyciformes) and a sister-group relationship between holocentrids and percomorphs, first recognized by Stiassny and Moore^75^ and Moore^76^, but challenged by Johnson and Patterson^4^.

Based on the topology obtained (Figs. 1-10, S1) we propose a new classification for ordinal and subordinal groups of bony fishes and subsequently discuss some of the most significant findings.

**Main phylogenetic hypothesis of bony fish groups collapsed to depict higher-level clades.**
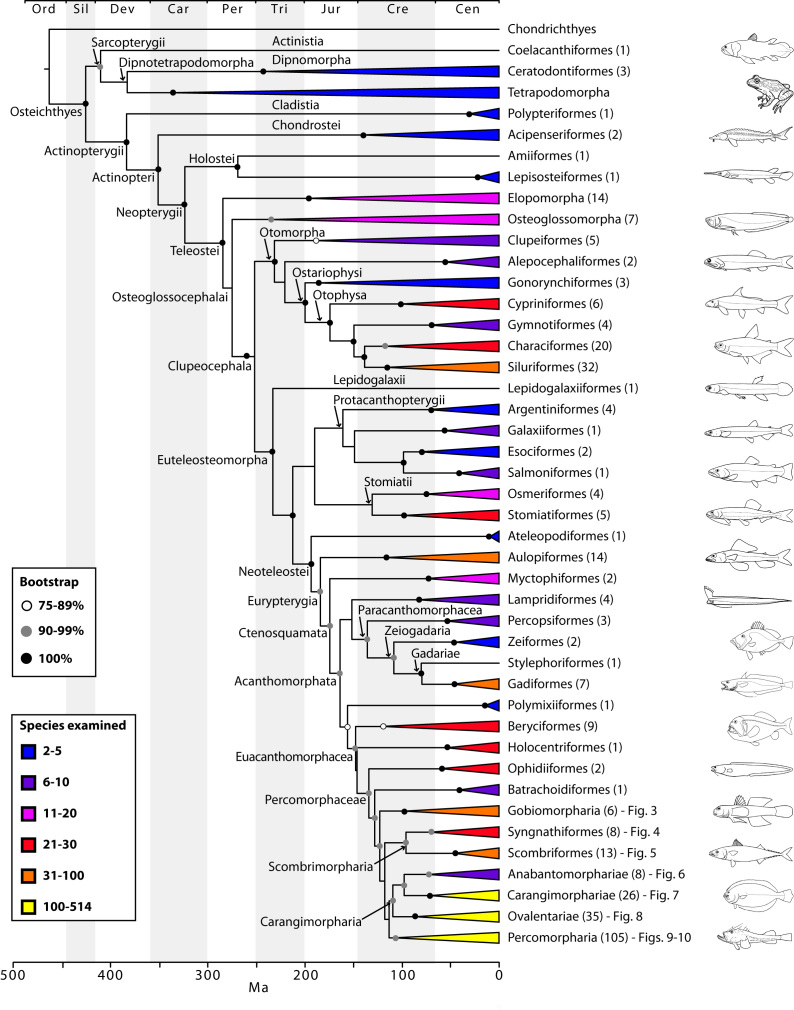
The phylogenetic tree was estimated in RAxML using the 3+ dataset (1416 taxa) and 24 partitions with divergence times estimated under PL using 126 fixed secondary calibrations from the BEAST analysis (see Fig. 11). Terminal clades are either orders or supraordinal taxa with multiple orders included. Values in parentheses indicate number of families examined. See also Figs. 3-10 for relationship details on selected percomorph clades. The complete phylogeny with bootstrap support values and names for supraordinal taxa is in Fig. S1).

**Table 2. Characterization of molecular markers examined.** *New markers developed here

**Locus**	**Description**	**Type**	**Total sequences**	**Alignment length (sites)**	**Pairwise identity (%)**
*16S*	16S rRNA	Mitochondrial	983	2326	70.0
*enc1*	Gene for peroxisomal enoyl-CoA hydratase/L-3-hydroxyacyl-CoA dehydrogenase	Nuclear exon	360	657	84.6
*ficd *	FIC domain	Nuclear exon	602	732	86.0
* gtdc2 *(*= glyt*)**	Glycosyltransferase-like domain containing 2	Nuclear exon	343	891	78.3
hoxc6a*	Homeo box C6a	Nuclear intron	362	1184	61.0
* kiaa1239*	Leucine-rich repeat and WD repeat-containing protein, KIAA1239-like	Nuclear exon	749	963	86.3
*myh6*	Myosin, heavy polypeptide 6	Nuclear exon	874	789	84.5
* panx2*	Pannexin 2	Nuclear exon	656	984	86.2
* plagl2*	Pleiomorphic adenoma gene-like 2	Nuclear exon	854	819	87.9
* ptchd1* (=*ptr*)	Patched domain containing 4	Nuclear exon	736	756	86.0
*rag1*	Recombination activating gene 1	Nuclear exon	784	1575	80.6
*rag2*	Recombination activating gene 2	Nuclear exon	276	1206	71.2
*rh*	Rhodopsin	Nuclear exon	417	927	84.2
*ripk4*	Receptor-interacting serine-threonine kinase 4	Nuclear exon	662	645	83.2
*snx33* (=*sh3px3*)	Sorting nexin 3; similar to SH3 and PX domain containing 3 gene	Nuclear exon	742	705	85.6
*sidkey *	si:dkey-174m14.3	Nuclear exon	547	1299	85.4
*gpr85 *(*=sreb2*)	G protein-coupled receptor 85	Nuclear exon	320	990	87.7
svep1*	Sushi, von Willebrand factor type A, EGF and pentraxin domain containing 1	Nuclear exon	226	825	77.6
*tbr1b* (=*tbr1*)	T-box, brain, 1b	Nuclear exon	601	831	86.4
vcpip*	Valosin-containing protein p97/p47 complete interacting protein 1	Nuclear exon	236	765	87.5
*zic1*	Zic family member 1	Nuclear exon	983	984	89.9

**Sensitivity analyses for selected clades obtained in this study (shown in Figs. 1, 3-10) and for selected alternative hypotheses.**
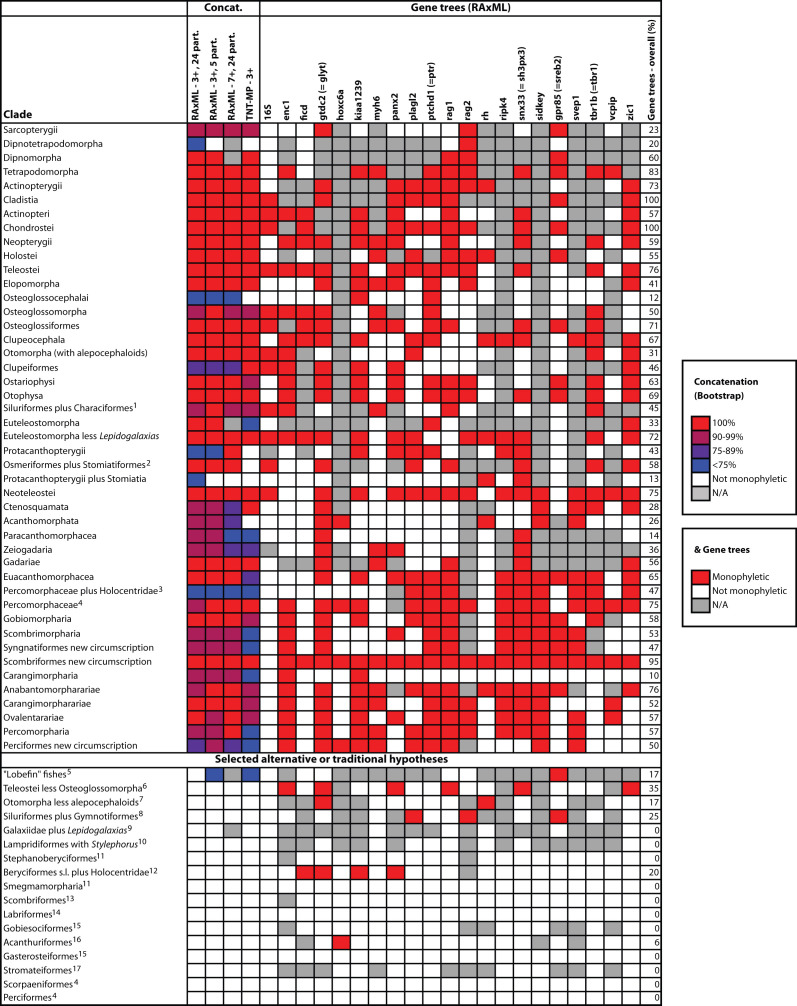
For each case, we assess support from individual gene trees (indicating whether the group was obtained) or from the concatenated data sets (indicating whether the group was obtained and showing boostrap support). For some gene trees, monophyletic groups ignore a few rogue taxa falling outside. N/A: insufficient taxonomic sampling to test hypothesis. ^1^Excluding Gymnotiformes (e.g., Saitoh et al.^77^); ^2^Stomiatii; ^3^Stiassny and Moore^75^; ^4^Nelson^2^ and Wiley and Johnson^5^; ^5^Shan and Gras^47^; ^6^Patterson and Rosen^78^; ^7^Arratia^79^; ^8^Fink and Fink^80^; ^9^Nelson^81^; ^10^Olney et al.^82^; ^11^Johnson and Patterson^4^; ^12^Miya et al.^8^; ^13^Johnson^83^; ^14^Kaufman and Liem^84^; ^15^Gill^85^; ^16^Acanthuriformes sensu Tyler et al.^86^ (i.e., Acanthuriformes sensu stricto^87^ plus Scatophagidae and Siganidae); ^17^Jordan^88^

**Revised Classification for Bony Fishes**

The nomenclatural arrangement presented in Appendix 2 builds on the existing classification by Wiley and Johnson^5^ and intends to preserve names and taxonomic composition of groups whenever possible. However, adjustments are made to recognize new well-supported molecular clades, many of which also have been obtained by previous molecular studies (several examples discussed below). Order-level or supraordinal taxa are erected (new) or resurrected on the basis of well-supported clades only (>90% bootstrap values). Current taxon names supported by previous molecular or morphological studies are retained if congruent with our results, even if bootstrap support is low (e.g., Osteoglossocephalai *sensu *Arratia^79^ with only 38% bootstrap). In some cases, ordinal or subordinal taxa that were not monophyletic in our analysis are also validated, as long as the incongruence is not supported by strong bootstrap values. Examples include the suborder Blennioidei (not monophyletic here but monophyletic in Wainwright et al.^31^) and the order Pleuronectiformes (not monophyletic here but monophyletic in Betancur-R. et al.^28^).

Family names for bony fishes are based on Eschmeyer and Fong^89^ and van der Laan et al.^90^, with minor modifications. Consult van der Laan et al.^90^ for authorship of family names and Wiley and Johnson^5^ for authorship of ordinal and subordinal names. Our list is not intended as a comprehensive revision of valid family names; instead, it is simply an adaptation of their list based on published studies that we know validate or synonymize family groups using explicit phylogenetic evidence. Unlike Eschmeyer and Fong^89^ and van der Laan et al.^90^, we do not recognize the family status of Anotopteridae, Omosudidae (synonyms of Alepisauridae^91^) or Latidae (synonym of Centropomidae^27^^,^ 92). Also, we recognize the following families, listed in Eschmeyer and Fong^89^ and van der Laan et al. 90 as synonyms or subfamilies of other families: Botiidae (following Chen et al.^93^), Diplophidae (following Nelson^2^; apparently omitted by Eschmeyer and Fong^89^), Horabagridae (following Sullivan et al.^94^), Sinipercidae (following Li et al.^96^), Steindachneriidae (following Roa-Varon and Ortí^98^), Zanclorhynchidae, the aulopiform Bathysauropsidae and Sudidae (following Davis^91^), and the pleuronectiform Paralichthodidae, Poecilopsettidae, and Rhombosoleidae (following Chapleau^97^, Munroe^99^, Betancur-R. et al.^28^). A total of 502 families are recognized here, of which 369 (73.5%) were examined. Of these, 146 families included only one representative (39.6%) and 40 (17.9%) of the remaining 223 were rendered non-monophyletic in our analysis (non-monophyletic families are indicated below). For each order/suborder we list all families examined as well as the unexamined families whose taxonomic affinity is expected on the basis of traditional taxonomy or phylogenetic evidence. The list of unexamined families is also intended as a resource that may help fish systematists to direct future sequencing efforts.

A total of 66 orders are classified, three of which are new (Holocentriformes, Istiophoriformes, and Pempheriformes), and 15 are resurrected or validated under a new circumscription. Some ordinal or subordinal names may appear to be new, but most can be found in the literature at various hierarchical levels. As examples, Spariformes is a Bleeker name and Centrarchiformes is a Webber and de Beaufort name. Because priority is not applied to names above the family level, we have not made a thorough attempt to establish first use. Only those three for which no reference could be found are listed as “new.” New infraorders are named in Suborder Cottioidei to circumscribe well-corroborated clades and may conserve the rank of superfamily in subsequent revisions. The ordinal status of 50 percomorph families examined (as well as many others unexamined) belonging to Carangimorphariae, Ovalentariae, and Percomorpharia remains uncertain (i.e.,*incertae sedis*) due to poor phylogenetic resolution. Percentages in parentheses following names indicate bootstrap support (no bootstrap values shown for redundant groups or monotypic taxa). The complete phylogenetic tree with annotated classification is illustrated in Fig. S1. The new classification scheme presented here should be considered a work in progress (version 1; Appendix 2), as any other hypothesis. It is likely to include involuntary errors and omissions in addition to the many unexamined, *sedis mutabilis,* and *incertae sedis* taxa. Updates should be forthcoming as new evidence become available and feedback from experts help refine it. For the most updated version visit DeepFin.

**Comparison of classifications**

Our results (Appendix 2) invite comparison to the recent classification of Wiley and Johnson^5^ based on morphological evidence gleamed from many investigators. Of 123 clades recognized by them, 70 (56.9%) are congruent with bootstrap values >95% obtained in this study. Five of these 70 clades are included in our sample by only one family and thus their monophyly is not critically tested. Another six clades (4.9%) are congruent but are supported by lower bootstrap values; seven additional clades (5.7%) are monotypic. Forty clades (32.5%) are incongruent, with some being grossly polyphyletic in our tree. Notable examples are Protacanthopterygii, Smegmamorpharia, and Labriformes. Others are incongruent based on exclusion of subclades and are rendered monophyletic in our classification by the addition or removal of smaller clades. Examples include Stomiatii (inclusion of Osmeriformes *sensu stricto*), Otomorpha (inclusion of Alepocephaliformes), Neoteleostei (removal of Stomiatiformes), and Lampridiformes (removal of *Stylephorus*).

There is considerable consensus between morphology and the interrelationships of major clades. For example, the major cohorts of living teleosts and their interrelationships are congruent with the listing convention employed by Wiley and Johnson^5^; this is also true within many of the major clades (e.g. relationships within Elopomorpha). But there is also incongruence. For example, relationships among early-branching acanthomorph groups differ considerably from previous morphological hypotheses (e.g., Johnson and Patterson^4^) with lampridiforms, percopsiforms, zeiforms and gadiforms branching off basally relative to polymixiiforms. More explicit tests of new and alternative phylogenetic hypotheses based on multiple analyses of our dataset are presented in Fig. 2.

**Novel Clades of Teleost Fishes**

The following sections highlight some of the salient features of this global phylogeny and classification of bony fishes, especially in reference to well-established relationships and newly found clades among the euteleosts. We do not attempt to provide a complete account of all taxonomic issues, but to give some perspective and contrast to discuss the evidence supporting novel and established taxa.

Early euteleost lineages: tenuous relationships (Fig. 1)

Our analyses support several recent hypotheses based on molecular data that contradict the consensus based on morphology^2^^,^ 5 relative to the composition of “protacanthopterygians.” Although our results fall short of resolving with confidence circumscription and relationships among taxa in this group (hence Protacathopterygii is a *sedis mutabilis* taxon in our proposed classification), some relationships are well supported and consistent with previous studies (Fig. 1). First, is the hypothesis that alepocephalid fishes (slickheads) have affinities within Otomorpha, instead of Argentiformes, as proposed by Johnson and Patterson^4^. This result was first proposed on the basis of mitogenomic data^10^^,^ 41 ^,^ 100 ^,^ 101 and recently corroborated with a subset of the nuclear markers used in this study^29^. Second, is the sister group relationship of Osmeriformes and Stomiatiformes (=Stomiiformes), first proposed by López et al.^21^ based on mtDNA and *rag1* sequence data. Finally, the position of *Lepidogalaxias* at the base of the euteleosts rendering Galaxiidae non-monophyletic also was proposed previously^102^^,^ 29 and supported by our data (see also Fig. 2).

*Paracanthomorphacea: mitogenomics dixit (Fig. 1)* **

This name was first introduced as superorder Paracanthopterygii (*sensu* Greenwood et al.^1^) to refer to a large group of spiny-finned fishes that included Batrachoidiformes, Gadiformes (with Ophioidei and Zoarcoidei), Gobiesociformes, Lophiiformes, and Percopsiformes. Many other taxa were added and also removed on the basis of conflicting evidence ever since Paracanthopterygii was conceived, but a conservative stance persisted in classifications supporting the original circumscription, with the exclusion of Gobiesociformes^2^. More recently, mitogenomic data^7^^,^ 8 discovered a sister-group relationship between Zeiformes and Gadiformes, a result also obtained with nuclear genes 19 ^,^ 24 ^,^ 103; the name Zeioigadiformes^24^ was coined for this new grouping. Miya et al.^11^ redefined the Paracanthopterygii to include Polymixiidae, Percopsiformes, Gadiformes, and Zeioidei and subsequently Miya et al.^13^ added to this group the lampridiform genus *Stylephorus*, which was unexpectedly found to form the sister group of Gadiformes. Analysis of four nuclear markers in addition to mtDNA confirmed this result^103^, supporting a monophyletic taxon Paracanthopterygii that includes percopsiforms, gadiforms, *Stylephorus* (placed in its own order Stylephoriformes) and zeiforms, in agreement with our results (Fig. 1, 2). A review of published morphological characters by Borden et al.^105^ also found significant congruence between this arrangement and morphological character-state distributions for many of the proposed relationships.

Euacanthomorphacea: holocentrids sister to percomorphs (Fig. 1)

Johnson and Patterson^4^ included polymixiids, percopsids and crown acanthomorphs in their Euacanthopterygii, a taxon not classified by Wiley and Johnson^5^. We adopt the name but modify the circumscription to recognize a well-supported clade (99% bootstrap) that includes beryciforms, holocentrids and percomorphs. The main issue at this level is delimitation of Beryciformes and relationships of its proposed components to Percomorphaceae. Most classifications^2^^,^ 4 accept separate orders Stephanoberyciformes and Beryciformes, each monophyletic and placed as successive sister-groups of the percomorphs. Molecular data (mitogenomic and smaller subsets of nuclear genes), in contrast, have supported the inclusion of Stephanoberyciformes in the same clade as Beryciformes^8^^,^ 29 and consistently include holocentrids within this clade. Our results, however, reject this hypothesis in favor of recognizing a separate holocentrid clade (proposed here as a new order, Holocentriformes) that is sister to percomorphs (Fig. 1), a result first obtained by Stiassny and Moore^75^ and Moore 76 but subsequently challenged by Johnson and Patterson^4^. Despite relatively low support for our holocentrid-percomorph clade (57-69% bootstrap), proportionally more individual gene trees support this relationship (47%) relative to the alternative molecular hypothesis uniting holocentrids with the remaining beryciform groups (20%; Fig. 2). Our new circumscription of Beryciformes is also most similar to that of the order Trachichthyiformes described by Moore^76^, except that the latter excludes the berycids.

 Percomorphaceae: no longer an unresolved bush (Figs. 1-10)

A major contribution from our study has been the disambiguation of the percomorph bush into nine well-supported supraordinal groups (six Series and three Subseries; Fig. 1; Appendix 2): Ophidiimorpharia, Batrachoidimorpharia, Gobiomorpharia (Fig. 3), Scombrimorpharia (Figs. 4 and 5), Carangimorpharia (with three Subseries: Anabantomorphariae, Fig. 6; Carangimorphariae, Fig. 7; and Ovalentariae, Fig. 8), and Percomorpharia (Figs. 9). Furthermore, increased phylogenetic resolution within Percomorpharia allowed the definition of a monophyletic Perciformes (Figs. 9 and 10), for the first time recovered from a vast taxonomic sample. With the exception of the cusk-eels (Ophidiimorpharia) and the toadfishes (Batrachoidimorpharia), whose monophyly has been recognized in most classifications (i.e., 2 ^,^ 5; but see 106 ^,^ 107), the remaining seven supraordinal clades (four Series and three Subseries) have never been discovered by examination of anatomical features. Under different combinations of taxa, however, and based on diverse genetic markers, several of these clades have been obtained, in one form or another, by previous molecular studies (e.g.,^7^^,^ 8 ^,^ 11 ^,^ 12 ^,^ 19 ^,^ 20 ^,^ 24 ^,^ 27 ^,^ 28 ^,^ 29 ^,^ 30 ^,^ 31 ^,^ 32 ^,^ 33).

A corollary of the increased resolution of percomorph relationships is the demise of the Smegmamorpharia *sensu* Johnson and Patterson^4^ (see also Wiley and Johnson^5^; Fig. 2). Elements included in this supraordinal taxon are now scattered throughout the molecular phylogeny, placed within many of the newly found clades with high bootstrap support. For example, the pygmy sunfishes (*Elassoma*) are back with the other sunfishes (centrarchids), as suggested by earlier classifications and recently confirmed by molecules^30^. Centrarchids plus elassomatids are placed here in the resurrected order Centrarchiformes (within Percomorpharia, Fig. 9). Mugiliforms (mullets) and atherinomorphs (silversides, needlefishes, halfbeaks, guppies and allies) are placed within Ovalentariae (Fig. 8). The swamp eels and spiny eels (order Synbranchiformes, suborders Synbranchoidei and Mastacembeloidei) are placed with confidence in Anabantomorphariae (Fig. 5), together with armored sticklebacks (Indostomidae), one of the 11 families previously included in the order Gasterosteiformes. The polyphyly of Gasterosteiformes (another large clade assigned to Smegmamorpha) was first pointed out by mitogenomic evidence^12^. Our results place the sticklebacks, tubesnouts and sand eels (previously assigned to Gasterosteoidei) in our newly defined Perciformes (suborder Cottioidei; Fig. 10) and the rest of the families previously assigned to the suborder Syngnathoidei were relocated to our newly defined order Syngnathiformes within the Scombrimorpharia (Fig. 4, see below).

Phylogenetic resolution within five newly discovered clades, however, will require additional study. Relationships within Syngnathiformes, Scombriformes, Carangimorphariae, Ovalentariae, and Percomorpharia may be challenging to recover given the rapid radiation and diversification of these clades.

*Gobiomorpharia: sweepers are out* *(Fig. 3)*

Based on a phylogeny estimated with four mitochondrial markers, Thacker^33^ resurrected the order Gobiiformes, to accommodate three suborders: Gobioidei (gobies and sleepers), Kurtidoidei (nurseryfish), and Apogonoidei (including apogonids and pempherids). Previous molecular studies have shown affinities between gobioids, apogonids, kurtids and, to some extent, pempherids and dactylopterids^8^^,^ 11 ^,^ 16. There is also morphological evidence supporting a close relationship between gobids and apogonids^108^^,^ 109 as well as between kurtids and apogonids^110^. Our results provide partial support for the Gobiiformes *sensu *Thacker^33^ but we treat it here as a supraordinal group (Gobiomorpharia). A major difference is that our hypothesis segregates the family Pempheridae (sweepers) to its own order (Pempheriformes, together with Glaucosomatidae), within Percomorpharia (Figs. 1, 3, 9).

**Detailed relationships among orders and families of Gobiomorpharia (see also Fig. 1).**
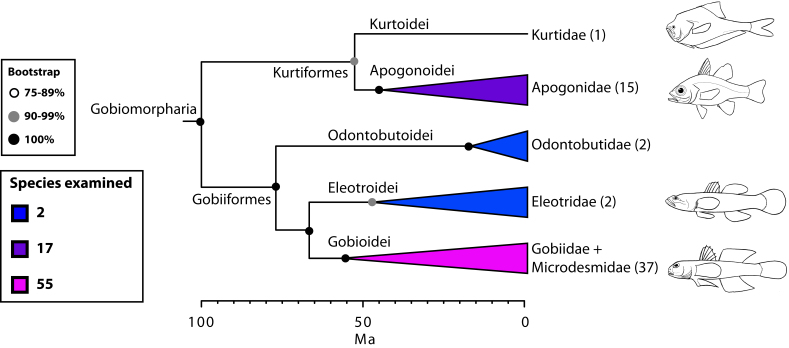
Values in parentheses indicate number of genera examined.

Scombrimorpharia: sea horses and tunas are close relatives (Figs. 1, 4 and 5)

One of the most unanticipated new percomorph clades is the Scombrimorpharia, grouping such disparate fishes as seahorses and tunas. This clade includes the newly circumscribed orders Syngnathiformes (Fig. 4) and Scombriformes (Fig. 5). Not surprisingly, a close relationship among taxa contained within this group, including syngnathids, mullids, callionymids, dactylopterids, scombrids, stromateids, an others, has never been proposed on morphological grounds. The Syngnathiformes, as defined here (Fig. 4), comprises mostly tropical marine reef-dwellers, traditionally placed in three distinct percomorph orders, including Gasterosteiformes (syngnathids), “Perciformes” (mullids and callionymids) and “Scorpaeniformes” (dactylopterids). Recent molecular studies have emphasized the non-monophyly of Scorpaeniformes^74^. We have noted above the dissolution of Gasterosteiformes^12^ and, as discussed below, we provide a restricted definition for Perciformes that includes many scorpaeniform taxa (Fig. 10).

**Detailed relationships among families of Syngnathiformes (see also Fig. 1).**
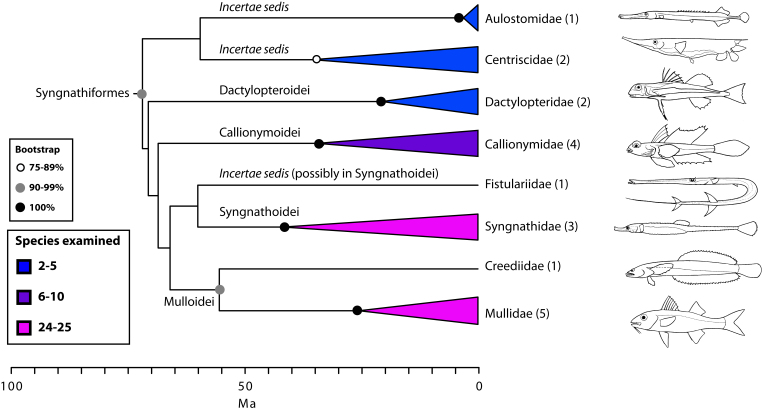
Values in parentheses indicate number of genera examined

Our new order Scombriformes (Fig. 5) includes most of the families previously grouped in the perciform suborder Scombroidei^2^ or the order Scombriformes^5^, except for the barracudas (Sphyraenidae) and the billfishes and swordfishes (here placed in their own order, Istiophoriformes). Sphyraenidae and Istiophoriformes are now firmly placed within Carangimorphariae (Fig. 7) together with disparate taxa such as remoras (Echeneidae), archer fishes (Toxotidae), jacks (Carangidae), flatfishes (Pleuronectiformes), and others (see below). Because billfishes and tunas are not closely related as previously suggested by anatomical studies^83^ (Fig. 2), the new hypothesis implies that endothermy has evolved at least twice independently in teleosts^111^^,^ 112. This new circumscription of Scombriformes also comprises families belonging to multiple orders in previous classifications, such as Stromateiformes (Centrolophidae, Nomeidae, Ariommatidae, Stromateidae), Trachiniformes (Chiasmodontidae), Icosteiformes (Icosteidae), and Perciformes (Bramidae, Pomatomidae, and Caristiidae). Despite the disparate morphology among members of Scombriformes, most are offshore fishes that inhabit pelagic and/or deep-sea waters.

**Detailed relationships among families of Scombriformes (see also Fig. 1).**
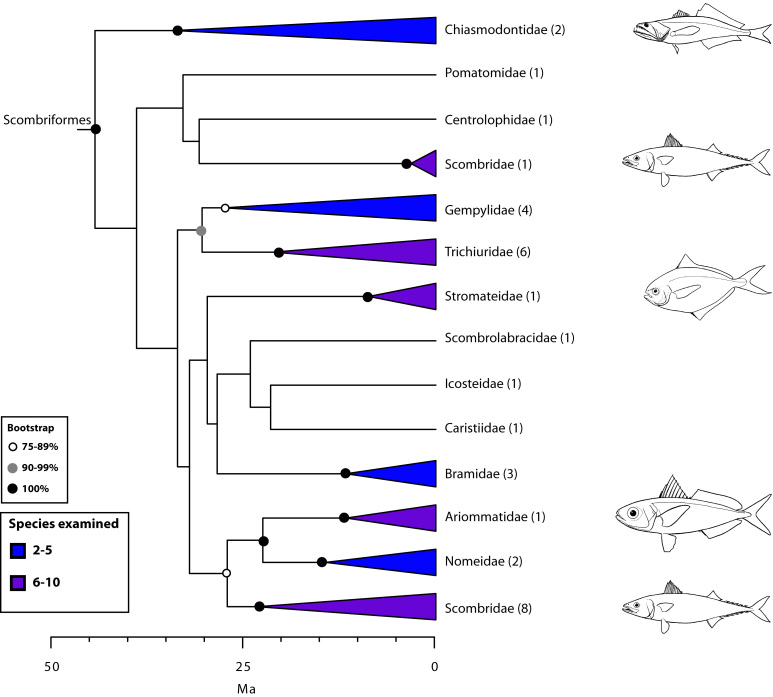
Values in parentheses indicate number of genera examined.

Anabantomorphariae: freshwater and air breathing (Fig. 6)

Another major percomorph group proposed here is the series Carangimorpharia, including three subseries: Anabantomorphariae, Carangimorphariae, and Ovalentariae (Fig. 1). Species in Anabantomorphariae include representatives placed in three separate orders by Wiley and Johnson^5^: Synbranchiformes (swamp eels), Gasterosteiformes (*Indostomus, *the armored stickleback), and Anabantiformes (gouramis) (Fig. 6). While the first two orders belonged to the Smegmamorpharia^4^^,^ 5, the Anabantiformes were placed as *incertae sedis *in Percomorphacea^5^. The monophyly of Anabantomorphariae has also been supported on the basis of mitogenomics^8^^,^ 11 ^,^ 12 and nuclear markers^28^. A remarkable condition shared by members of this novel grouping is their mostly freshwater origin and restriction to Africa and South East Asia (although some members in the family Synbranchidae occur in Mexico, and Central and South America). Most are able to occupy marginal, stagnant waters due to their capacity to tolerate anoxia and to obtain oxygen directly from the air. Anabantiforms have a suprabranchial organ and synbranchids have suprabranchial pouches with respiratory function.

**Detailed relationships among orders and families of Anabantomorphariae (see also Fig. 1).**
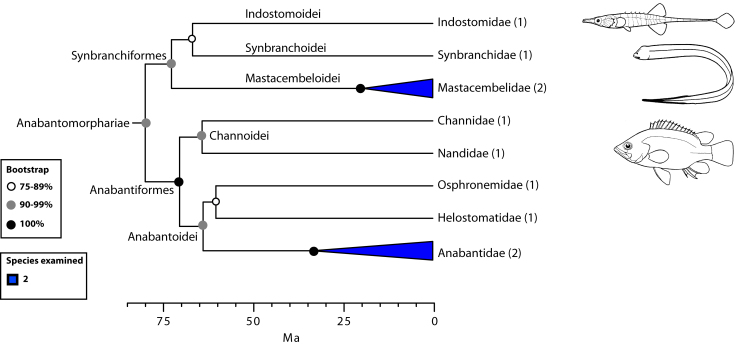
Values in parentheses indicate number of genera examined.

Carangimorphariae: flatfishes and unlikely relatives (Fig. 7)

A close affinity between other seemingly disparate groups, including barracudas, swordfishes, jacks, flatfishes, and others, has been well established by recent molecular studies^10^^,^ 16 ^,^ 19 ^,^ 24 ^,^ 27 ^,^ 28 ^,^ 112 (Fig. 7). This higher-level group has been referred to as ‘‘clade L’’ sensu Chen et al.^19^ or Carangimorpha by Li et al.^24^ (see also^27^^,^ 28). In looking for possible anatomical synapomorphies uniting flatfishes, billfishes, and carangids, Little et al.^112^ found that most taxa share a relatively low number of vertebrae, have multiple dorsal pterygiophores inserting before the second neural spine, and lack supraneurals, among others. However, according to Friedman^113^, some of these characters are symplesiomorphies while others are absent in the remaining carangimorph groups. It thus seems paradoxical that despite the apparent lack of morphological synapomorphies for carangimorphs there is a strong molecular signal supporting their monophyly, whereas the opposite is true for pleuronectiforms^28^. For additional insights and discussion on Carangimorphariae we refer the reader to recent studies^24^^,^ 27 ^,^ 28 ^,^ 112 ^,^ 113.

**Detailed relationships among orders and families of Carangimorphariae (see also Fig. 1).**
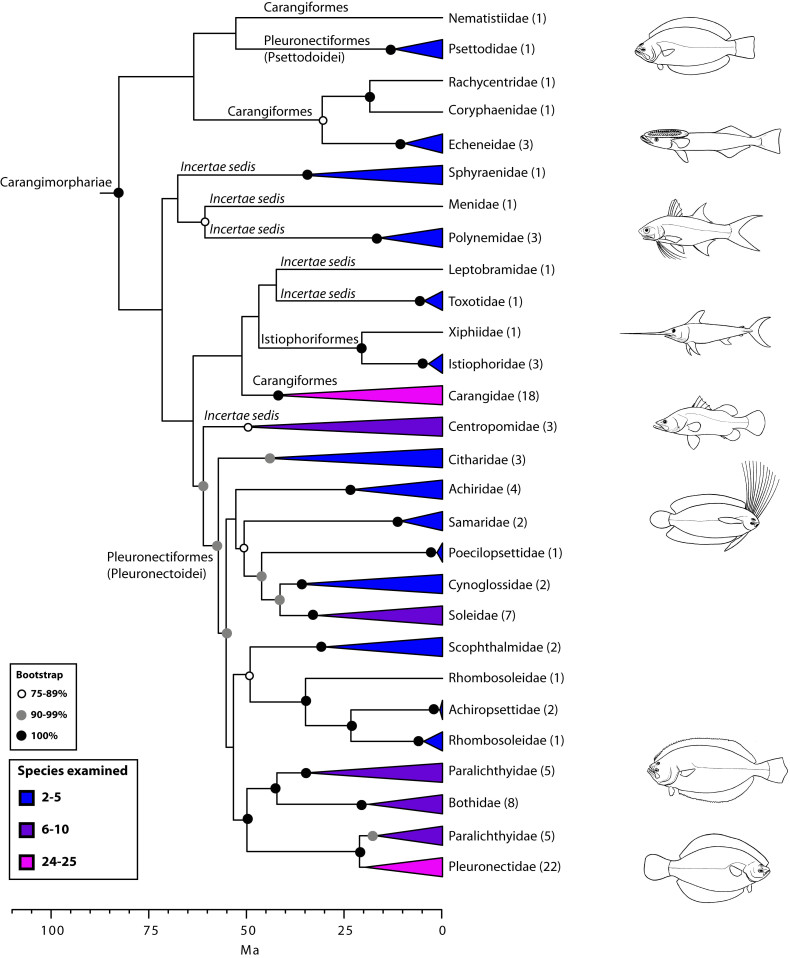
Values in parentheses indicate number of genera examined (see also Betancur-R. et al.^28^).

Ovalentariae: sticky eggs (Fig. 8)

Ovalentariae is one of the most spectacular percomorph radiations, including more than 5000 species in some 44 families, grouping seemingly distinct groups such as cichlids, mullets, blennies, and atherinomorphs (atheriniforms, beloniforms, and cyprinodontiforms). This clade was first found on the basis of mitogenomic evidence^8^^,^ 12 and later confirmed with nuclear sequence data^23^^,^ 24 ^,^ 26 ^,^ 31. Our results suggest that this group can be divided into four subgroups (superorders), two of which already existed (Atherninomorphae and Mugilomorphae) and two that are new: (i) Cichlomorphae (Cichlidae plus Pholidichthyidae) and (ii) Blennimorphae (blennioids plus clingfishes, jawfishes and basslets). Many families in Ovalentariae, however, remain *incertae sedis *(e.g., Embiotocidae and Pseudochromidae). Two different studies have coined a name for this group; first Stiassnyiformes **by Li et al.^24^ and, more recently, Ovalentaria by Wainwright et al.^31^ for their characteristic demersal, adhesive eggs with chorionic filaments (lost secondarily in some groups). An interesting implication of this phylogenetic hypothesis is that the pharyngeal jaw apparatus (pharyngognathy), present in many members of this clade (e.g., Cichlidae, Pomacentridae, Hemiramphidae), has evolved multiple times in percomorphs^31^. We refer the reader to Wainwright et al.^31^ for additional discussion on Ovalentariae.

**Detailed relationships among orders and families of Ovalentariae (see also Fig. 1).**
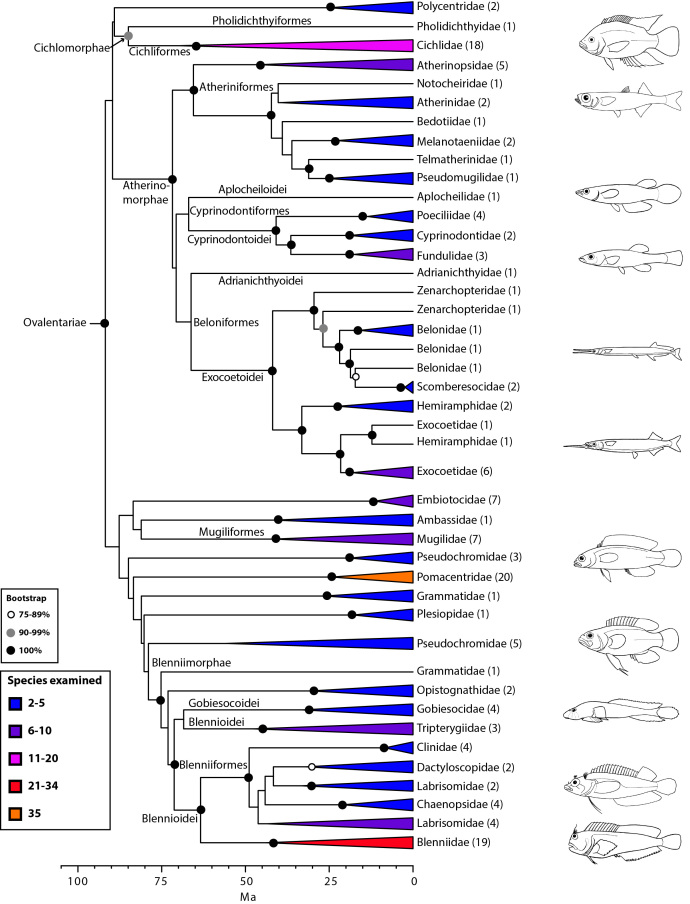
Values in parentheses indicate number of genera examined (see also Wainwright et al. 31). Many clades lacking taxonomic annotations on nodes are incertae sedis taxa (for details, see classification).

*Percomorpharia: the new bush at the top* *(Fig. 9)*

Percomorpharia is by far the largest percomorph clade, including 11 orders with some of the most prominent ones such as Perciformes, Labriformes, Lophiiformes, and Tetraodontiformes. At least 151 families (105 examined) belong in Percomorpharia, including three of the top ten most diverse families of fishes (i.e., Labridae, Serranidae, and Scorpaenidae)^2^. More than one third (514) of the species in our bony fish phylogeny are placed in this clade. Previous molecular studies obtained monophyletic groups with a combination of taxa here assigned to Percomorpharia, but with far more limited sampling (e.g., 8 ^,^ 11 ^,^ 16 ^,^ 74). Although most family-level and ordinal groups within Percomorpharia receive high bootstrap support, interrelationships among them are largely unresolved (hence, the new bush at the top; Fig. 9). Several of these groups are newly proposed or resurrected orders under new circumscription (e.g., Uranoscopiformes, Ephippiformes, Pempheriformes). Our new arrangement removes anglerfishes (Lophiiformes) from Paracanthomorphacea, as was suggested by previous classifications^78^, and places them close to tetraodontiforms, caproids, acanthuriforms, chaetodontids, pomacanthids, ephippids and others (see also 87 ^,^ 114 ^,^ 115). The largest group within Percomorpharia is the order Perciformes.

**Detailed relationships among orders and families of Percomorpharia (the new bush at the top; see also Fig. 1).**
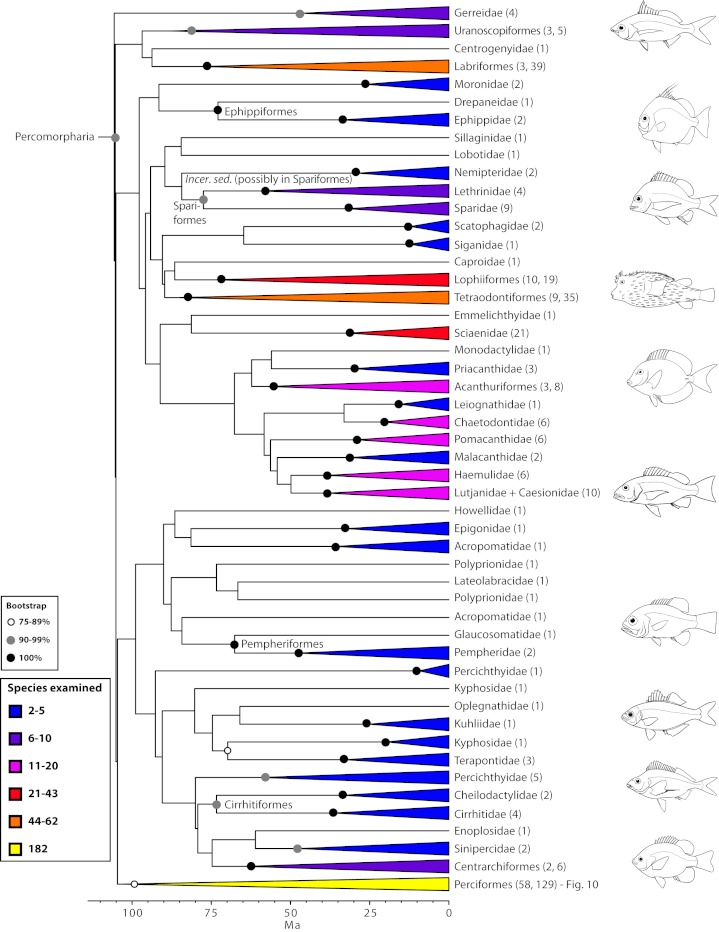
Values in parentheses indicate number of genera examined in each terminal family or number of families and genera, respectively, in each terminal order. See also Fig. 10 for expanded relationships on perciform groups. Many clades lacking taxonomic annotations on nodes are incertae sedis taxa (for details, see classification).

*Perciformes: no longer a taxonomic waste basket* *(Fig. 10)*

For the first time, a monophyletic definition of Perciformes can be recovered from phylogenetic analysis of a comprehensive taxon sampling. The new circumscription of Perciformes reduces significantly the number of included taxa, while retaining remarkable diversity that can be organized into several suborders and infraorders. Nelson’s classification^2^ included 160 families in Perciformes, making it the largest order of all vertebrates. Our definition indicates unambiguous membership for 38 families and uncertain membership for an additional 42 that were not examined in our study but that have been assigned to either “Perciformes” (10), “Scorpaeniformes” (14), Cottiformes (8), or Trachiniformes (1) in previous classifications^2^^,^ 5. Hence, the maximum possible number of families in the newly defined Perciformes is reduced to 71. This number is closer to the 90 families proposed by Wiley and Johnson^5^ for their Perciformes, but with a very different composition.

For a long time, Perciformes has been regarded as a “taxonomic waste basket”^2^^,^ 5 with ‘‘percoids’’ scattered throughout Percomorpha and no clear phylogenetic distinction among Percoidei, Perciformes, and Percomorpha^74^. Earlier molecular studies lacked sufficient sampling to resolve phylogenetic questions among “percoids,” but close relationships among groupers (Serranidae), perches (Percidae), sticklebacks (Gasterosteidae), searobins (Triglidae), icefishes (Notothenioidei), sculpins (Cottoidei), eelpouts (Zoarcoidei) and scorpionfishes (Scorpaenoidei) have been obtained in one form or another, and in different combinations, by several authors^16^^,^ 19 ^,^ 20 ^,^ 23 ^,^ 24 ^,^ 29 ^,^ 74 ^,^ 116. All of these taxa are included in our definition of Perciformes (Fig. 10).

Within Perciformes, we tentatively propose suborders (Notothenioidei, Scorpaenoidei, Trigloidei, Cottoidei) for clades with high support that also represent some well-established groups, but two *incertae sedis* (Percophidae and Platycephalidae), and several unexamined families remain unclassified. Additional taxon sampling and more data are needed to resolve interrelationships among these taxa. Four suborders/infraorders were recognized as separate orders by Wiley and Johnson^5^: Percoidei, Scorpaenoidei, Cottioidei, and Gasterosteales (an infraorder of Cottioidei).

The composition of Perciformes obtained from our phylogeny is remarkably similar to a group named “Serraniformes” by Li et al.^24^. This choice of name is misleading, given that Percidae is included and serranids have historically been considered a family within Perciformes. Adoption of Serraniformes would obliterate the long ichthyological tradition of defining higher taxa with the prefix “perco” for hierarchical groups that contain perciforms (preserved in our classification). Most recently, the same team of researchers (Lautredou et al.^116^) presented a detailed analysis of this clade using seven nuclear markers and obtained phylogenetic relationships that are generally congruent with our results (Fig. 10), albeit they support a close relationship of Percophidae with notothenioids and divide platycephaloids into three groups. We refer the reader to this paper, as well as others (e.g., Smith and Wheeler^16^; Smith and Craig^74^), for more details on taxonomic issues.

**Detailed relationships among families of Perciformes (see also Figs. 1 and 9).**
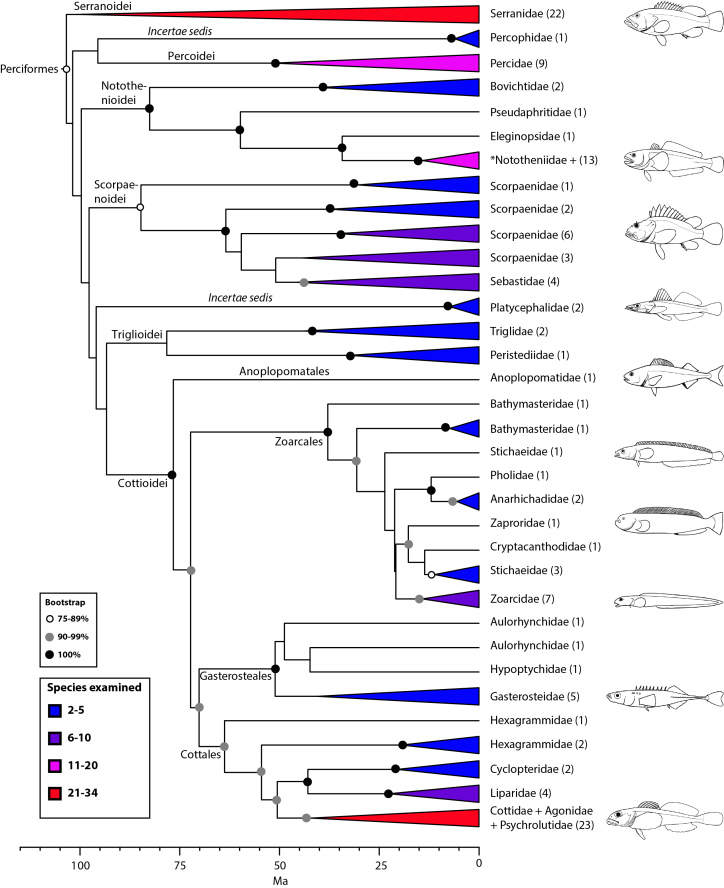
Values in parentheses indicate number of genera examined. *Nototheniidae sensu lato, including the families Nototheniidae sensu stricto, Artedidraconidae, Harpagiferidae, Bathydraconidae, and Channichthyidae.

**A New Timescale of Bony Fish Evolution**

In addition to the novel insights regarding the interrelationships of teleost fishes, our study provides the most comprehensively sampled time-tree of bony fish evolution based on 60 calibrations points (Figs. 1, 11). Recent studies that estimated divergence times using multi-locus nuclear approaches had more restricted taxonomic focus and implemented fewer (<36) fossil calibrations^29^^,^ 66. The time-calibrated phylogeny for bony fishes provided here should stimulate macroevolutionary studies of fishes using phylogenetic comparative methods (PCMs).

**Time-calibrated BEAST phylogeny based on a subset of 202 taxa, indicating the placement for the 59 calibrations used.**
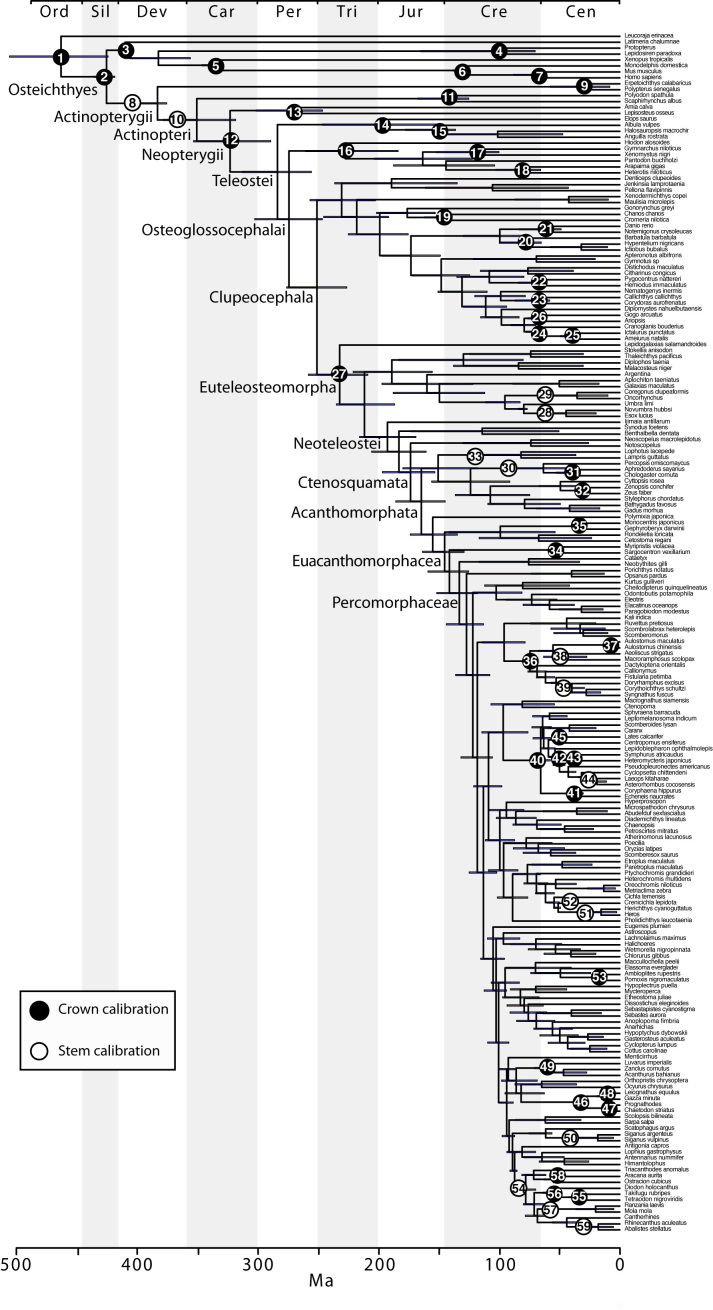
Bars represent the 95% highest posterior credibility intervals of divergence times. Calibration (60) was not included for this analysis (see Appendix 1).

Although our approach for calibrating the molecular phylogeny is based on a set of common fossil constraints used by the cited studies and others^64^^,^ 65, some differences in the results are evident (Fig. 12). Our estimates of mean divergence dates for early actinopterygian lineages tend to be younger and the divergence of neoteleosts and subtending clades are substantially older relative to those in Near et al.^29^ (although 95% probability distributions overlap in many cases). Sensitivity of molecular calibrations to different combinations of taxa, molecular markers, and fossil constraints have been discussed extensively by several authors, suggesting that development of better statistical methods and best practice approaches should decrease disparity among estimated ages of clades^43^^,^ 118 ^,^ 119. Compared to earlier studies reporting divergence times among teleost lineages^35^^,^ 36 ^,^ 37 ^,^ 120 ^,^ 121 ^,^ 41, estimates based on multi-locus data and denser taxonomic sampling tend to converge (Fig. 11), suggesting that the current trend to analyze larger data sets with well established fossil constraints will result in robust time trees in the future.

**Comparison of mean (triangle) and 95% highest posterior credibility intervals (horizontal bars) of divergence dates for selected clades (see also Figs 1, 11).**
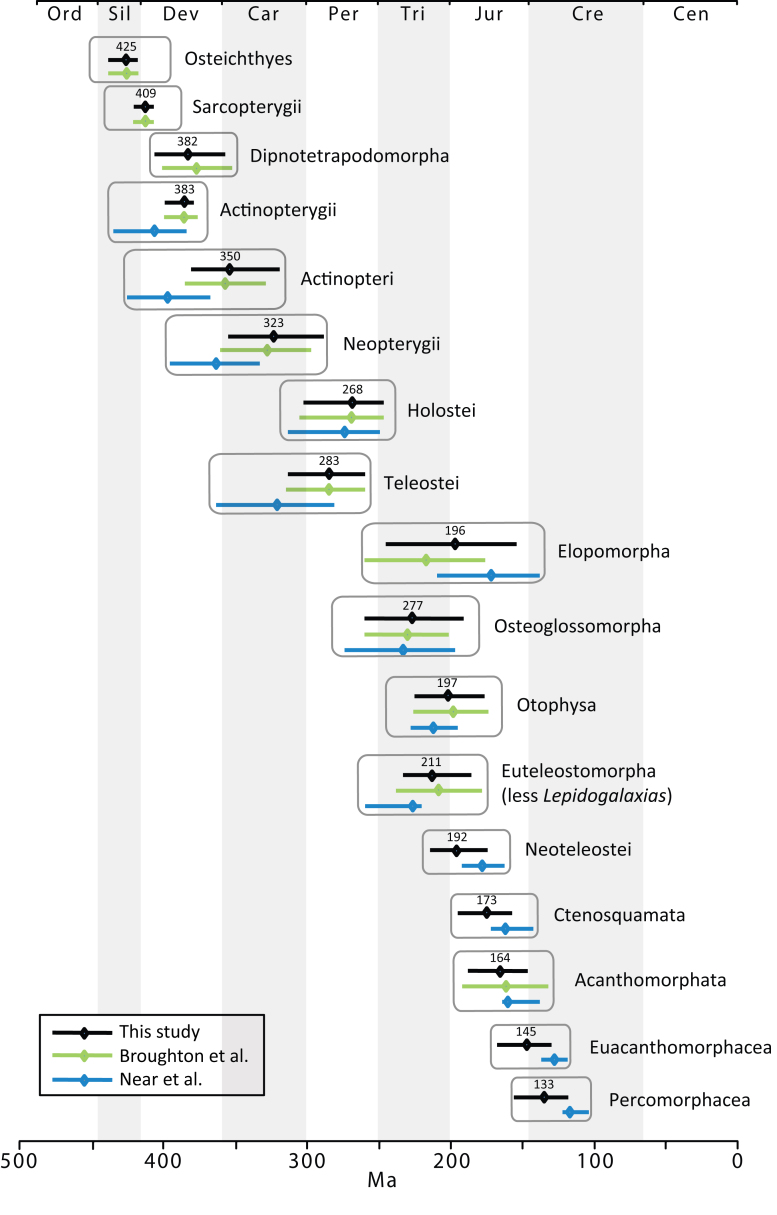
Black lines and mean dates are from this study, blue are from Near et al.^29^ and green are from Broughton et al.^66^ Absent lines imply that the particular date estimation was not performed in the corresponding study.

The date estimates presented herein (Figs 1, 2-11, and the OneZoom tree) confirm the notion that divergences of major ray-finned fish lineages are considerably older than the oldest known fossils for their respective groups^29^^,^ 66. Our estimate of 425 Ma for divergence of crown Osteichthyes places the origin of Sarcopterygii and Actinopterygii in the Middle Silurian, with the sarcopterygian crown group evolving in the Early Devonian (409 Ma) and the actinopterygian crown group evolving at the Middle-Late boundary of the Devonian (383 Ma), both of which correspond to the “Age of Fishes”. Although the oldest teleost fossils are from the late Triassic (e.g., †*Pholidophorus latiusculus*, Norian^122^), the molecular hypothesis suggests that the initial divergence of crown group Teleostei occurred long before in the Early Permian (283 Ma). Appearance of the three major teleost lineages (Elopomorpha, Osteoglossomorpha and Clupeocephala) took place in a narrow temporal window of 13 million years during this period. Paleoecological conditions surrounding the end Permian mass extinction, which resulted in extinction of up to 96% of all marine species of that time^123^, might have shaped the evolutionary history of early teleosts.

Recent work suggested that a major burst of teleost diversification, predominantly within Otophysa and Percomorphacea, took place in a relatively short time span between the late Mesozoic and early Cenozoic^29^^,^ 124. Patterns in the fossil record corroborate this idea, revealing an explosive morphological diversification of percomorphs in the aftermath of the end-Cretaceous extinction^125^. According to our estimates, however, the major lineages within Percomorphaceae (Ophidiiformes, Batrachoidiformes, Gobiomorpharia, Scombrimorpharia, Carangimorpharia, Percomorpharia and Perciformes) originated between 132 Ma and 82 Ma, before the end of the Cretaceous. The same is true for the diversification of many lineages within each of these groups, but explicit analyses using robust PCMs would be necessary to assess rate shifts of lineage diversification through time.

**Remaining Challenges and Unresolved Issues**

The new shape of the tree of life of bony fishes and the classification reflecting this structure offered by this study leaves many questions unanswered and suggests several directions for future sequencing efforts. Many families not included in the present analysis are listed in the classification and many groups defined as *incertae sedis* or *sedis mutabilis *clearly deserve additional study. Relationships for many terminal taxa, such as those within the rapid percomorph radiations, are often poorly resolved, have low bootstrap support, or have dubious resolution due to the combination of missing data, taxon sampling, and or other sources of systematic error. The relatively high proportion of missing data in the 3+ dataset (59%) is likely to have a stronger topological impact at the fine scale (towards the tips); e.g., two sister taxa with little or no genetic overlapping may not be resolved as closely related. Another major factor that may severely compromise phylogenetic inference is compositional heterogeneity (non-stationarity), in particular for gene trees, as suggested by a recent study that examined a fraction of the taxa and markers included here^28^. Unfortunately, efficient non-stationary approaches to analyze large and heterogeneous multi-locus data sets such as the one presented here currently are not available. Fish orders with dubious internal relationships include the Characiformes, Gymnotiformes, Lophiiformes, Pleuronectiformes, Carangiformes, among others.

Several parts of the fish tree that require additional study include (i) resolution of the relationships among coelacanths, lungfishes and tetrapods^46^^,^ 47 ^,^ 66; (ii) the basal divergence of euteleosteomorph groups and circumscription of Protacanthopterygii, in particular interrelationships of argentiniforms, galaxiiforms, osmeriforms, salmoniforms, esociforms, stomiatiforms and neoteleosts; (iii) interrelationships among components within Scombrimorpharia, Carangimorphariae, Ovalentariae, Percomorpharia, and Perciformes; and (iv) the ordinal status of 55 percomorph families examined (as well as many others unexamined) that remain with uncertain ordinal affiliation (*incertae sedis*). We predict that these difficult challenges in ichthyology will find renewed sources of evidence with the advent of next generation sequencing approaches and phylogenomics (e.g., 126 ^,^ 127). Reinterpretation of morphology and new studies of developmental patterns will be necessary to reconcile the molecular phylogenetic hypothesis with existing and expanding phenotypic data sets (e.g., 128 ^,^ 129).

## Author contributions

CL and GO designed the molecular markers; REB, GO, RBR, KC, JAL, CL, EOW, TG, NIH, KL, TG, and DA designed the taxonomic sampling; GL, DJH, and TL digitized the EToL database; CL, MS, JC, RBR, FZ, TB, MC, DA, ARV, SW, and WCB generated sequence data via PCR and edited chromatograms; RBR and DA downloaded sequences from NCBI, vetted all the sequences compiled, and assembled the datasets; RBR and JAB performed phylogenetic analyses; RBR and GA selected the fossil calibrations and RBR estimated divergence times; RBR, DA, REB made the figures; RBR and EOW proposed the new classification and NIH, GA, GO, and DA made insightful additions; RBR and GO wrote the paper and RB, GO and GA took care of the final editing; all authors contributed to the writing.

## Appendix 1

### Calibration points and age prior settings

This section provides details on calibrations and prior settings used for divergence time estimates. Hard lower bounds or minimum age reflect the youngest possible age interpretation of fossils, rather than mid-point of age range [see 1]; soft upper bounds or maximum age indicate the oldest possible fossil age. One geological calibration uses a normal distribution with soft minimum and maximum bounds (calibration 37). For calibration details, refer to the original studies cited under each; prior distributions and settings for some calibrations were adapted for this study.

(1) Gnathostomata. MRCA: *Leucoraja, Danio*. Hard minimum age: 426.0 Ma. 95% soft maximum age: 519.0 Ma. Prior setting: Lognormal distribution, mean= 6.15, St. Dev.= 0.06 (crown calibration). Calibration source: Broughton et al. [2].

(2) Osteichthyes. MRCA: *Latimeria, Danio*. Hard minimum age: 418.0 Ma. 95% soft maximum age: 438.0 Ma. Prior setting: Exponential distribution, mean= 6.66 (crown calibration). Calibration source: Broughton et al. [2].

(3) Sarcopterygii. MRCA: *Latimeria, Lepidosiren*. Hard minimum age: 407.0 Ma. 95% soft maximum age: 419.0 Ma. Prior setting: Exponential distribution, mean= 4.00 (crown calibration). Calibration source: Broughton et al. [2].

(4) Lepidosirenoidei. MRCA: *Lepidosiren, Protopterus*. Hard minimum age: 70.0 Ma. 95% soft maximum age: 416.0 Ma. Prior setting: Uniform distribution (crown calibration). Calibration source: Broughton et al. [2].

(5) Tetrapoda. MRCA: *Xenopus, Homo*. Hard minimum age: 330.0 Ma. 95% soft maximum age: 350.0 Ma. Prior setting: Exponential distribution, mean= 6.69 (crown calibration). Calibration source: Broughton et al. [2], Benton and Donoghue [3].

(6) Metatheria. MRCA: *Monodelphis, Homo*. Hard minimum age: 124.5 Ma. 95% soft maximum age: 139.0 Ma. Prior setting: Exponential distribution, mean= 4.50 (crown calibration). Calibration source: Broughton et al. [2], Benton and Donoghue [3].

(7) Human-Mouse. MRCA: *Mus, Homo*. Hard minimum age: 62.0 Ma. 95% soft maximum age: 101.0 Ma. Prior setting: Exponential distribution, mean= 12.84 (crown calibration). Calibration source: Broughton et al. [2], Benton and Donoghue [3].

(8) Actinopterygii (total group). MRCA: *Polypterus, Danio*. Hard minimum age: 398.0 Ma. 95% soft maximum age: 423.0 Ma. Prior setting: Exponential distribution, mean= 8.35 (stem calibration). Calibration source: Broughton et al. [2].

(9) Polypteriformes. MRCA: *Erpetoichthys, Polypterus*. Hard minimum age: 5.0 Ma. 95% soft maximum age: 99.0 Ma. Prior setting: Uniform distribution (crown calibration). Calibration source: Broughton et al. [2].

(10) Actinopteri (total group). MRCA: *Scaphirhynchus, Danio*. Hard minimum age: 375.0 Ma. 95% soft maximum age: 415.0 Ma. Prior setting: Exponential distribution, mean= 13.34 (stem calibration). Calibration source: Broughton et al. [2].

(11) Chondrostei. MRCA: *Scaphirhynchus, Polyodon*. Hard minimum age: 125.0 Ma. 95% soft maximum age: 246.0 Ma. Prior setting: Exponential distribution, mean= 40.40 (crown calibration). Calibration source: Broughton et al. [2].

(12) Neopterygii. MRCA: *Lepisosteus, Danio*. Hard minimum age: 260.0 Ma. 95% soft maximum age: 386.0 Ma. Prior setting: Uniform distribution (crown calibration). Calibration source: Broughton et al. [2].

(13) Holostei. MRCA: *Lepisosteus, Amia*. Hard minimum age: 246.0 Ma. 95% soft maximum age: 350.0 Ma. Prior setting: Exponential distribution, mean= 34.70 (crown calibration). Calibration source: Broughton et al. [2].

(14) Elopomorpha. MRCA: *Elops, Halosauropsis*. Hard minimum age: 149.0 Ma. 95% soft maximum age: 260.0 Ma. Prior setting: Uniform distribution (crown calibration). Calibration source: Broughton et al. [2].

(15) Albuliformes + Anguilliformes. MRCA: *Albula, Halosauropsis*. Hard minimum age: 136.0 Ma. 95% soft maximum age: 216.0 Ma. Prior setting: Exponential distribution, mean= 26.70 (crown calibration). Calibration source: Broughton et al. [2].

(16) Osteoglossomorpha. MRCA: *Hiodon, Arapaima*. Hard minimum age: 130.0 Ma. 95% soft maximum age: 260.0 Ma. Prior setting: Uniform distribution (crown calibration). Calibration source: Broughton et al. [2].

(17) Notopteridae (total group). MRCA: *Gymnarchus, Xenomystus*. Hard minimum age: 100.0 Ma. 95% soft maximum age: 216.0 Ma. Prior setting: Exponential distribution, mean= 38.73 (crown calibration). Calibration source: Broughton et al. [2].

(18) Arapaimidae. MRCA: *Arapaima, Heterotis*. Hard minimum age: 65.5 Ma. 95% soft maximum age: 136.0 Ma. Prior setting: Exponential distribution, mean= 23.55 (crown calibration). Calibration source: Broughton et al. [2].

(19) Chanidae. MRCA: *Chanos, Cromeria*. Hard minimum age: 139.0 Ma. 95% soft maximum age: 216.0 Ma. Prior setting: Exponential distribution, mean= 25.70 (crown calibration). Calibration source: Broughton et al. [2]. Comments: Kneriidae is not represented in the taxonomic sampling of Broughton et al. [2]; modified to include the MRCA of Chanidae plus Kneriidae.

(20) Cobitoidea. MRCA: *Hypentelium, Barbatula*. Hard minimum age: 60.0 Ma. 95% soft maximum age: 146.5-145.5 Ma, stem ostariophysan †*Tischlingerichthys viohli*, Late Jurassic, Germany [4]. Prior setting: Exponential distribution, mean= 27.20 (crown calibration). Calibration source: Saitoh et al. [5] (prior setting adapted for this study).

(21) Cyprinidae. MRCA: *Danio, Notemigonus*. Hard minimum age: 48.5 Ma. 95% soft maximum age: 146.5-145.5 Ma, stem ostariophysan †*Tischlingerichthys viohli* (see calibration 20 above). Prior setting: Exponential distribution, mean= 32.70 (crown calibration). Calibration source: Saitoh et al. [5] (prior setting adapted for this study).

(22) Serrasalmidae + Hemiodontidae. MRCA: *Pygocentrus, Hemiodus*. Hard minimum age: 61.0 Ma. 95% soft maximum age: 97.0 Ma. Prior setting: Exponential distribution, mean= 12.02 (crown calibration). Calibration source: Broughton et al. [2].

(23) Callichthyidae. MRCA: *Callichthys, Corydoras*. Hard minimum age: 58.0 Ma. 95% soft maximum age: 146.5-145.5 Ma, stem ostariophysan †*Tischlingerichthys viohli*, Late Jurassic, Germany. Prior setting: Exponential distribution, mean= 29.53 (crown calibration). Calibration source: Lundberg et al. [6] (prior setting adapted for this study).

(24) Ictaluridae + Cranoglanidae. MRCA: *Ictalurus, Cranoglanis*. Hard minimum age: 63.0 Ma. 95% soft maximum age: 146.5-145.5 Ma, stem ostariophysan †*Tischlingerichthys viohli* (see calibration 20 above). Prior setting: Exponential distribution, mean= 27.87 (crown calibration). Calibration source: Lundberg et al. [6] (prior setting adapted for this study).

(25) Ictaluridae. MRCA: *Ameiurus, Ictalurus*. Hard minimum age: 34.0 Ma. 95% soft maximum age: 63.0 Ma, stem ostariophysan †*Tischlingerichthys viohli* (see calibration 20 above). Prior setting: Exponential distribution, mean= 10.01 (crown calibration). Calibration source: Lundberg et al. [6] (prior setting adapted for this study).

(26) Arioidea. MRCA: *Gogo, Ariopsis*. Hard minimum age: 65.5 Ma. 95% soft maximum age: 146.5 Ma. Prior setting: Exponential distribution, mean= 27.05 (crown calibration). Calibration source: Broughton et al. [2].

(27) Euteleostei. MRCA: *Lepidogalaxias, Takifugu*. Hard minimum age: 149 Ma. 95% soft maximum age: 260 Ma. Prior setting: Uniform distribution (crown calibration). Calibration source: Broughton et al. [2].

(28) Esocidae + Umbridae. MRCA: *Esox, Novumbra*. Hard minimum age: 76.5 Ma. 95% soft maximum age: 87.5 Ma. Prior setting: Lognormal distribution, mean= 1.09, St. Dev.= 0.8 (stem calibration). Calibration source: Near et al. [7].

(29) Salmonidae. MRCA: *Coregonus, Oncorhynchus*. Hard minimum age: 51.8 Ma. 95% soft maximum age: 76.4 Ma. Prior setting: Lognormal distribution, mean= 1.62, St. Dev.= 0.8 (stem calibration). Calibration source: Near et al. [7].

(30) Percopsidae. MRCA: *Percopsis, Aphredoderus*. Hard minimum age: 57.0 Ma. 95% soft maximum age: 65.5 Ma. Prior setting: Lognormal distribution, mean= 0.53, St. Dev.= 0.8 (stem calibration). Calibration source: Near et al. [7].

(31) Aphredoderidae. MRCA: *Aphredoderus, Chologaster*. Hard minimum age: 34.0 Ma. 95% soft maximum age: 59.0 Ma. Prior setting: Lognormal distribution, mean= 1.90, St. Dev.= 0.8 (crown calibration). Calibration source: Near et al. [7].

(32) Zenopsis + Zeus. MRCA: *Zenopsis, Zeus*. Hard minimum age: 32 Ma. 95% soft maximum age: 36.5 Ma. Prior setting: Lognormal distribution, mean= 0.23, St. Dev.= 0.8 (crown calibration). Calibration source: Near et al. [7].

(33) Lampridiformes. MRCA: *Lophotus, Lampris*. Hard minimum age: 56 Ma. 95% soft maximum age: 83.5 Ma. Prior setting: Lognormal distribution, mean= 2.01, St. Dev.= 0.8 (stem calibration). Calibration source: Near et al. [7]. Comments: *Lophotus* was not examined by Near et al. [7]; modified to include the MRCA of *Lophotus* and *Lampris*.

(34) Holocentridae. MRCA: *Myripristis, Sargocentron*. Hard minimum age: 50 Ma. 95% soft maximum age: 57.5 Ma. Prior setting: Lognormal distribution, mean= 0.67, St. Dev.= 0.8 (crown calibration). Calibration source: Near et al. [7]. Comments: Near et al. [7] report the fossil as a stem lineage of Myripristinae and assigns it to the divergence of Myripristinae + Holocentrinae. However, their figure S2 shows the calibration placed in the stem of Holocentridae; it should be the crown.

(35) Trachichthyidae. MRCA: *Gephyroberyx, Monocentris*. Hard minimum age: 32 Ma. 95% soft maximum age: 36.5 Ma. Prior setting: Lognormal distribution, mean= 0.23, St. Dev.= 0.8 (crown calibration). Calibration source: Near et al. [7].

(36) Syngnathiformes (*sensu lato*). MRCA: *Aulostomus, Callionymus*. Hard minimum age: 70.5 Ma. 95% soft maximum age: 81 Ma. Prior setting: Lognormal distribution, mean= 1.02, St. Dev.= 0.8 (crown calibration). Calibration source: Near et al. [7].

(37) *Aulostomus* - Panama Isthmus. MRCA: *A. maculatus, A. chinensis*. 5% soft minimum age: 2.8 Ma. 95% soft maximum age: 3.5 Ma. Prior setting: Normal distribution, mean= 3.15, St. Dev.= 0.212 (crown calibration). Calibration source: Bowen et al. [8].

(38) Centriscidae (total group). MRCA: *Aeoliscus, Macroramphosus*. Hard minimum age: 50 Ma. 95% soft maximum age: 57.5 Ma. Prior setting: Lognormal distribution, mean= 0.67, St. Dev.= 0.8 (stem calibration). Calibration source: Near et al. [7]. Comments: Near et al. [7] used this as crown calibration one node below (with *Aulostomus*). Instead, we place it in the stem Centriscidae because the position of this group is unstable.

(39) Syngnathidae (total group). MRCA: *Syngnathus, Doryrhamphus*. Hard minimum age: 50 Ma. 95% soft maximum age: 57.5 Ma. Prior setting: Lognormal distribution, mean= 0.67, St. Dev.= 0.8 (stem calibration). Calibration source: Near et al. [7]. Comments: Near et al. [7] noted that there is no phylogenetic resolution for *†*“*Syngnathus*”* heckeli *and *†Prosolenostomus lessinii* among syngnathids and assigned these fossils as stem calibration points for the MRCA of *Fistularia *and *Syngnathus*. However, because *Fistularia* is not a syngnathid (i.e., lacks body plates), a more appropriate placement for these two fossils (both of which have body plates) is the stem Syngnathidae.

(40) Carangiformes. MRCA: *Caranx, Echeneis*. Hard minimum age: 56 Ma. 95% soft maximum age: 64 Ma. Prior setting: Lognormal distribution, mean= 0.78, St. Dev.= 0.8 (crown calibration). Calibration source: Near et al. [7].

(41) Echeneidae + Coryphaenidae + Rachycentridae. MRCA: *Coryphaena, Echeneis*. Hard minimum age: 30 Ma. 95% soft maximum age: 34.5 Ma. Prior setting: Lognormal distribution, mean= 0.17, St. Dev.= 0.8 (crown calibration). Calibration source: Near et al. [7].

(42) Pleuronectoidei. MRCA: *Lepidoblepharon, Heteromycteris*. Hard minimum age: 50 Ma. 95% soft maximum age: 57.5 Ma. Prior setting: Lognormal distribution, mean= 0.67, St. Dev.= 0.8 (crown calibration). Calibration source: Near et al. [7].

(43) Soleidae + Cynoglossidae. MRCA: *Heteromycteris, Symphurus*. Hard minimum age: 40.5 Ma. 95% soft maximum age: 50 Ma. Prior setting: Lognormal distribution, mean= 0.95, St. Dev.= 0.8 (crown calibration). Calibration source: Near et al. [7]. Comments: Near et al. [7] placed the calibration one node below (Samaridae + Soleidae + Cynoglossidae) arguing that "Chanet (1994) argues that †*Eobuglossus* can be identified as a soleid on the basis of the geometry of the ascending process of the blind side premaxilla. We are not convinced that the state in this fossil can be meaningfully distinguished from the condition found in cynoglossids." A placement †*Eobuglossus* in the Soleidae + Cynoglossidae crown reconciles both opinions.

(44) Bothidae (total group). MRCA: *Asterorhombus, Laeops*. Hard minimum age: 30 Ma. 95% soft maximum age: 34.5 Ma. Prior setting: Lognormal distribution, mean= 0.17, St. Dev.= 0.8 (stem calibration). Calibration source: Near et al. [7]. Comments: Near et al. [7] assigned the fossil **†** *Oligobothus* to the divergence of Bothidae + Pleuronectidae; it is likely a stem bothid [9].

(45) Centropomidae (new crown calibration). MRCA: *Centropomus, Lates*. Hard minimum age: †*Eolates gracilis* [10] and †*Centropomus* [11]. Diagnosis and phylogenetic placement: placement of †*Eolates gracilis* in Latinae is supported by the presence of posterior pad in infraorbital 1 and by having 10+14 vertebrae; †*Eolates gracilis* is the earliest branching lineage of Latiinae [10]. Stratigraphic horizon and locality: early Eocene, upper Ypresian, Monte Bolca, Italy. Absolute age estimate: 50 Ma [12]. 95% soft upper bound: 57.5 Ma, based on the FA95 (Marshall [13]; following Near et al. [7]). Prior setting: Lognormal distribution, mean= 0.67, St. Dev.= 0.8.

(46) Chaetodontidae (total group). MRCA: *Chaetodon, Leiognathus*. Hard minimum age: 30 Ma. 95% soft maximum age: 34.5 Ma. Prior setting: Lognormal distribution, mean= 0.17, St. Dev.= 0.8 (crown calibration). Calibration source: Near et al. [7].

(47) *Chaetodon* + *Prognathodes*. MRCA: *Chaetodon, Prognathodes*. Hard minimum age: 7.1 Ma. 95% soft maximum age: 9 Ma. Prior setting: Lognormal distribution, mean= 0.10, St. Dev.= 0.3 (crown calibration). Calibration source: Near et al. [7].

(48) Leiognathidae. MRCA: *Gazza, Leiognathus*. Hard minimum age: 11.5 Ma. 95% soft maximum age: 23 Ma. Prior setting: Lognormal distribution, mean= 1.12, St. Dev.= 0.8 (crown calibration). Calibration source: Near et al. [7].

(49) Luvaridae. MRCA: *Luvarus, Acanthurus*. Hard minimum age: 56 Ma. 95% soft maximum age: 64 Ma. Prior setting: Lognormal distribution, mean= 0.78, St. Dev.= 0.8 (crown calibration). Calibration source: Near et al. [7].

(50) Siganidae (total group). MRCA: *S. argenteus, S. vulpinus*. Hard minimum age: 56 Ma. 95% soft maximum age: 64 Ma. Prior setting: Lognormal distribution, mean= 0.78, St. Dev.= 0.8 (stem calibration). Calibration source: Near et al. [7].

(51) Cichlidae - Heroini. MRCA: *Heros, Herichthys*. Hard minimum age: 49 Ma. 95% soft maximum age: 143 Ma. Prior setting: Exponential distribution, mean= 31.40 (stem calibration). Calibration source: López-Fernández et al. [14]. Comments: a stem calibration is required because our taxonomic sampling lacks early-branching heroins.

(52) Cichlidae - Geophagini. MRCA: *Crenichichla*. Hard minimum age: 49 Ma. 95% soft maximum age: 143 Ma. Prior setting: Exponential distribution, mean= 31.40 (terminal-branch calibration). Calibration source: López-Fernández et al. [14].

(53) *Ambloplytes *+* Pomoxis*. MRCA: *Ambloplytes, Pomoxis*. Hard minimum age: 15.5 Ma. 95% soft maximum age: 18 Ma. Prior setting: Lognormal distribution, mean= 0.10, St. Dev.= 0.5 (crown calibration). Calibration source: Near et al. [7]. Comments: Near et al. [7] assign †*Archoplites* to the divergence of *Archoplites* + *Ambloplytes*. Because our taxonomic sampling lacks *Archoplites*, we assign it to the MRCA of *Ambloplytes *+* Pomoxis*, following Collar et al. [15] (i.e., *Pomoxis* + *Archoplites* + *Ambloplytes*).

(54) Tetraodontiformes (total group). MRCA: *Triacanthodes, Takifugu*. Hard minimum age: 85 Ma. 95% soft maximum age: 122 Ma. Prior setting: Exponential distribution, mean= 11.69 (stem calibration). Calibration source: Alfaro et al. [16], Alfaro et al. [17]. Comments: Alfaro et al. [17] and Santini et al. [18] argue: "The oldest taxon assigned to this clade is the stem tetraodontiform †*Cretatriacanthus guidottii* from the Santonian of Nardo (Italy). We chose this taxon to date the minimum age rather than other, older [†*Plectocretacicus clarae*], stem tetraodontiformes because preliminary reexamination of the relationships of extant and fossil tetraodontiforms (Santini, unpublished) casts doubt on their phylogenetic affinities." Although we adopt Santini’s interpretation here, these results should be taken cautiously until explicit evidence challenging the traditional placement of †*Plectocretacicus* (e.g., [19]) becomes available.

(55) Tetraodontidae. MRCA: *Takifugu, Tetraodon*. Hard minimum age: 32 Ma. 95% soft maximum age: 50 Ma. Prior setting: Exponential distribution, mean= 6.01 (crown calibration). Calibration source: Alfaro et al. [16], Alfaro et al. [17]. Comments: The fossils used by Near et al. [7] are stem diodontids. While they argue that their assignment is one node below (i.e., divergence of Tetraodontidae + Diodontidae), their figure S2 shows the calibration placed in the stem of this clade; it should be the crown.

(56) Diodontidae + Tetraodontidae. MRCA: *Diodon, Tetraodon*. Hard minimum age: 50 Ma. 95% soft maximum age: 85 Ma (stem †*Cretatriacanthus*). Prior setting: Exponential distribution, mean= 11.52 (crown calibration). Calibration source: Alfaro et al. [16] (prior setting adapted for this study).

(57) Molidae (total group). MRCA: *Mola, Ranzania*. Hard minimum age: 41 Ma. 95% soft maximum age: 85 Ma (stem †*Cretatriacanthus*). Prior setting: Exponential distribution, mean= 14.52 (stem calibration). Calibration source: Alfaro et al. [16] (prior setting adapted for this study).

(58) Aracanidae + Ostraciidae. MRCA: *Aracana, Ostracion*. Hard minimum age: 50 Ma. 95% soft maximum age: 85 Ma (stem †*Cretatriacanthus*). Prior setting: Exponential distribution, mean= 11.52 (crown calibration). Calibration source: Alfaro et al. [16] (prior setting adapted for this study).

(59) Balistidae (total group). MRCA: *Rhinecanthus, Abalistes*. Hard minimum age: 35 Ma. 95% soft maximum age: 85 Ma (stem †*Cretatriacanthus*). Prior setting: Exponential distribution, mean= 16.52 (stem calibration). Calibration source: Alfaro et al. [16] (prior setting adapted for this study).

(60) Chondrichthyes (new crown calibration). MRCA: *Leucoraja, Callorhinchus*. Hard minimum age: †*Chondrenchelys problematica *and †*Onychoselache*. Diagnosis and phylogenetic placement: †*Chondrenchelys *is the earliest unambiguous fossil holocephalan as supported by several synapomorphies: holostylic jaw suspension; precerebral fontanelle absent; orbit separated from nasal capsule by broad, laterally facing orbitonasal lamina; Meckel's cartilage fused at symphysis; tooth families reduced, among others [20]. †*Onychoselache* is the oldest stem-group elasmobranch; details of the jaws, braincase and postcranial skeleton demonstrate that Onychoselache is a well-characterized member of the Hybodontiformes [21]. Stratigraphic horizon and locality: both †*Chondrenchelys* and †*Onychoselache* are from the Visean (early Mississippian), Glencartholm, Scotland. Absolute age estimate: 338 Ma. 95% soft maximum age: 438 Ma, **†** *Elegestolepis conica*, oldest stem chondrichthyan, Middle-Late Llandovery, Angara-Jlim area, Niuya River outcrops and Niuya-Berresova area, Siberian Platform [22]. Prior setting: Exponential distribution, mean= 33.39. Comment: implementation of this calibration was suggested by M. Coates (pers. comm.) after completion of the BEAST analyses. Therefore, it is only used for the PL analysis of the complete tree, as hard minimum and hard maximum age constrains. Although not implemented here, the priors proposed are intended as a reference for future studies. ****

**References**

1. Parham JF, Donoghue PC, Bell CJ, Calway TD, Head JJ, et al. (2012) Best practices for justifying fossil calibrations. Systematic Biology 61: 346-359.

2. Broughton RE, Betancur-R. R, Li C, Arratia G, Orti G (2013) Multi-locus phylogenetic analysis reveals the pattern and tempo of bony fish evolution. PLOS Currents Tree of Life.

3. Benton MJ, Donoghue PCJ (2007) Paleontological evidence to date the tree of life. Molecular Biology and Evolution 24: 26-53.

4. Arratia G (1997) Basal teleosts and teleostean phylogeny. Palaeo Ichthyologica 7: 1-168.

5. Saitoh K, Sado T, Doosey MH, Bart Jr HL, Inoue JG, et al. (2011) Evidence from mitochondrial genomics supports the lower Mesozoic of South Asia as the time and place of basal divergence of cypriniform fishes (Actinopterygii: Ostariophysi). Zoological Journal of the Linnean Society 161: 633-662.

6. Lundberg JG, Sullivan JP, Rodiles-Hernández R, Hendrickson DA (2007) Discovery of African roots for the Mesoamerican Chiapas catfish, *Lacantunia enigmatica*, requires an ancient intercontinental passage. Proceedings of the Academy of Natural Sciences of Philadelphia 156: 39-53.

7. Near TJ, Eytan RI, Dornburg A, Kuhn KL, Moor JA, et al. (2012) Resolution of ray-finned fish phylogeny and timing of diversification. Proceedings of the National Academy of Sciences 109: 13698–13703.

8. Bowen BW, Bass AL, Rocha LA, Grant WS, Robertson DR (2001) Phylogeography of the trumpetfishes (Aulostomus): ring species complex on a global scale. Evolution 55: 1029-1039.

9. Baciu DS, Chanet B (2002) The fossil flatfishes (Teleostei: Pleuronectiformes) from the Oligocene of Piatra Neamt (Romania). Oryctos 4: 17–38.

10. Otero O (2004) Anatomy, systematics and phylogeny of both Recent and fossil latid fishes (Teleostei, Perciformes, Latidae). Zoological Journal of the Linnean Society 141: 81-133.

11. Sepkoski JJJ (2002) A compendium of fossil marine animal genera. Bulletins of American Paleontology 363: 1-560.

12. Andrea Papazzoni C, Trevisani E (2006) Facies analysis, palaeoenvironmental reconstruction, and biostratigraphy of the “Pesciara di Bolca” (Verona, northern Italy): An early Eocene Fossil-Lagerstätte. Palaeogeography, Palaeoclimatology, Palaeoecology 242: 21-35.

13. Marshall CR (2008) A simple method for bracketing absolute divergence times on molecular phylogenies using multiple fossil calibration points. American Naturalist 171: 726-742.

14. López-Fernández H, Arbour JH, Winemiller KO, Honeycutt RL (2013) Testing for ancient adaptive radiations in Neotropical cichlid fishes. Evolution: in press.

15. Collar DC, O'Meara BC, Wainwright PC, Near TJ (2009) Piscivory limits diversification of feeding morphology in centrarchid fishes. Evolution; international journal of organic evolution 63: 1557-1573.

16. Alfaro ME, Santini F, Brock CD (2007) Do Reefs Drive Diversification in Marine Teleosts? Evidence from the Pufferfish and Their Allies (Order Tetraodontiformes). Evolution 61: 2104-2126.

17. Alfaro ME, Faircloth BC, Sorenson L, Santini F (2012) A phylogenomic perspective on the radiation of ray-finned fishes based upon targeted sequencing of ultraconserved elements. arXiv.

18. Santini F, Sorenson L, Marcroft T, Dornburg A, Alfaro ME (2013) A multilocus molecular phylogeny of boxfishes (Aracanidae, Ostraciidae; Tetraodontiformes). Molecular Phylogenetics and Evolution 66: 153-160.

19. Tyler JC, Sorbini L (1996) New superfamily and three new families of tetraodontiform fishes from the Upper Cretaceous: the earliest and most morphologically primitive plectognaths. Smithsonian Contributions to Paleobiology 82: 1–59.

20. Stahl BJ (1999) Chondrichthyes III: Holocephali. In: Schultze H-P, editor. Handbook of Paleoichthyology: Verlag Dr. Friedrich Pfeil. pp. 164.

21. Coates MI, Gess RW (2007) A new reconstruction of *Onychoselache traquairi*, comments on early chondrichthyan pectoral girdles and hybodontiform phylogeny. Palaeontology 50: 1421–1446.

22. Karatajüte-Talimaa V (1995) The Mongolepidae: Scale structure and systematics position. Geobios 19: 35-37.

## Appendix 2

### Revised Classification for Bony Fishes

Download PDF
